# Supplementary material for: Elucidating the protein interaction network of one of the largest icosahedral capsids in the virosphere
Source: EMBO J. 2026 Apr 10;45(10):3514–39. doi: 10.1038/s44318-026-00770-8 (PMC13186993; doi:10.1038/s44318-026-00770-8)
Supplement: Supplementary file 3 — Table EV3 [file 44318_2026_770_MOESM3_ESM.pdf]

Table EV3: List of primers used in this study.

Primer ID	Purpose	Sequence
HS1	5' homology arm F for recombination L515 (HA tag)	CTTTTGCAAAAAGCTTTATTAAATATAATTTGACTAATCC
**Primer ID**	**Purpose**	**Sequence**
HS1	5' homology arm F for recombination L515 (HA tag)	CTTTTGCAAAAAGCTTTATTAAATATAATTTGACTAATCC
HS2	5' homology arm R for recombination L515 (HA tag)	AACATCGTATGGGTAATAATTTCTACGATTATTTTTAATC
HS3	3' homology arm F for recombination L515 (HA tag)	AAATAGTCCTTTAGAATTTATCCCACAAATAAATTATCA
HS4	3' homology arm R for recombination L515 (HA tag)	CTTATCGCTGCGGCCGCAATTCCAACAATGTTATGAATAAAC
HS5	5' homology arm F for recombination L330 (HA tag)	CTTTTGCAAAAAGCTTATATTTATGGAATGATGGATCC
HS6	5' homology arm R for recombination L330 (HA tag)	AACATCGTATGGGTATGGGAAAGAATAAATTTTATATAA
HS7	3' homology arm F for recombination L330 (HA tag)	AAATAGTCCTTTAGATATTGTTTTTTTTAATCGAGTTT
HS8	3' homology arm R for recombination L330 (HA tag)	CTTATCGCTGCGGCCGCCTGAAATAGAAACTCTTGCA
HS9	5' homology arm F for recombination R710 (HA tag)	CTTTTGCAAAAAGCTTGTGTCACAACAATCCAAT
HS10	5' homology arm R for recombination R710 (HA tag)	AACATCGTATGGGTATTTTTCTGATTTATACAACCA
HS11	3' homology arm F for recombination R710 (HA tag)	AAATAGTCCTTTAGATCATTCATTGAAAAATTTATTAAT
HS12	3' homology arm R for recombination R710 (HA tag)	CTTATCGCTGCGGCCGCTCTAAAATCAATAGGACAATCG
HS13	5' homology arm F for recombination R443 (HA tag)	CTTTTGCAAAAAGCTTCTATAGATATAAATTATGCGTAAAC
HS13	5' homology arm R for recombination R443 (HA tag)	AACATCGTATGGGTATTGATTCATGTTAGATCGG
HS14	3' homology arm F for recombination R443 (HA tag)	AAATAGTCCTTTAGATCGACAAATATACTAGTCAAATAAT
HS15	3' homology arm R for recombination R443 (HA tag)	CTTATCGCTGCGGCCGCCAAGGACAGTGGGAGAAAT
HS16	5' homology arm F for recombination R387 (HA tag)	CTTTTGCAAAAAGCTTAACAGAAATGCATTGGATC

HS17	5' homology arm R for recombination R387 (HA tag)	AACATCGTATGGGTAATGGATATTAACTGAATTGATTCTG
HS18	3' homology arm F for recombination R387 (HA tag)	AAATAGTCCTTTAGAAATACAAATCAACCAATTTAGTG
HS19	3' homology arm R for recombination R387 (HA tag)	CTTATCGCTGCGGCCGCATTCAGATAAAAAATTTTGGAT
HS20	5' homology arm F for recombination L274 (HA tag)	CTTTTGCAAAAAGCTTATCTCAGCACTCTCTACCATAATTC
HS21	5' homology arm R for recombination L274 (HA tag)	AACATCGTATGGGTAGGGGAGAGGTTCAAGTTGATC
HS22	3' homology arm F for recombination L274 (HA tag)	AAATAGTCCTTTAGAATTTCTAGACATTTTAACCCC
HS23	3' homology arm R for recombination L274 (HA tag)	CTTATCGCTGCGGCCGCAAATTCACCATTAGCAATAAAATCC
HS24	5' homology arm F for recombination R347 (HA tag)	CTTTTGCAAAAAGCTTTACAGTAAACTAGTTCCCGTT
HS25	5' homology arm R for recombination R347 (HA tag)	AACATCGTATGGGTAAATAGATTGCAAATATCGTGTTTTT
HS26	3' homology arm F for recombination R347 (HA tag)	AAATAGTCCTTTAGATAGATGTTTTTTTTGGTTTATTCTC
HS27	3' homology arm R for recombination R347 (HA tag)	CTTATCGCTGCGGCCGCTCATTATTTCCAACACCA
HS28	5' homology arm F for recombination R287(HA tag)	CTTTTGCAAAAAGCTTATTGTATGGAGGTTTCGAAAAAC
HS29	5' homology arm R for recombination R287(HA tag)	AACATCGTATGGGTATTGACCAGTTAAATAAATTCCAG
HS30	3' homology arm F for recombination R287 (HA tag)	AAATAGTCCTTTAGATTTTGTCTAATATTTTATCTAAT
HS31	3' homology arm R for recombination R287 (HA tag)	CTTATCGCTGCGGCCGCAATCCATCTTCTGATAATAGT
HS32	5' homology arm F for recombination R513b (HA tag)	CTTTTGCAAAAAGCTTCCATACGATTTAGAGGAACAAGT
HS33	5' homology arm R for recombination R513b (HA tag)	AACATCGTATGGGTATAAGAAACTGGCATTCCTGTTTTTG
HS34	3' homology arm F for recombination for R513b (HA tag)	AAATAGTCCTTTAGAAAATTGATTGAATAACCT
HS35	3' homology arm R for recombination R513b (HA tag)	CTTATCGCTGCGGCCGCGATTTGTCATATTAATTATTCT

HS36	5' homology arm F for recombination L567 (HA tag)	CTTTTGCAAAAAGCTTATAGTGGACAATGGGTTGGATGG
HS37	5' homology arm F for recombination L567 (HA tag)	AACATCGTATGGGTATTGAGCACCTAATTGTTCTAATCTT
HS38	3' homology arm F for recombination L567 (HA tag)	AAATAGTCCTTTAGAATTTATTTTTTCCAATTAAATTTG
HS39	3' homology arm R for recombination L567 (HA tag)	CTTATCGCTGCGGCCGCCCAAAAGAATTTTGAAATGAG
HS40	5' homology arm F for recombination L487 (HA tag)	CTTTTGCAAAAAGCTTACAAAATATTAAACCTAATAATAT
HS41	5' homology arm R for recombination L487 (HA tag)	AACATCGTATGGGTATTTTTTAACGACAGAATTTCT
HS42	3' homology arm F for recombination L487 (HA tag)	AATAGTCCTTTAGATTTAATAGATCGTAATAAATTAACT
HS43	3' homology arm R for recombination L487 (HA tag)	CTTATCGCTGCGGCCGCCTCTAGTACCATTACCAGCTG
HS44	5' homology arm F for recombination L323 (HA tag)	CTTTTGCAAAAAGCTTTCAACAATACTCAAAATCTAATTTC
HS45	5' homology arm R for recombination L323 (HA tag)	AACATCGTATGGGTAATATATTCTATTAGAATCATTTGGA
HS46	3' homology arm F for recombination L323 (HA tag)	AATAGTCCTTTAGATTGACTAGTATAAACTGGATATTT
HS47	3' homology arm R for recombination L323 (HA tag)	CTTATCGCTGCGGCCGCGTCAAAATATGAACAAATGGAT
HS48	5' homology arm F for recombination R335 (HA tag)	CTTTTGCAAAAAGCTTCCTAATCAATATAATCAAAACAATC
HS49	5' homology arm R for recombination R335 (HA tag)	AACATCGTATGGGTAAACTGAATTTCCCACAAATC
HS50	3' homology arm F for recombination R335 (HA tag)	AATAGTCCTTTAGACAATATACCAATAAAAAAATTG
HS51	3' homology arm R for recombination R335 (HA tag)	CTTATCGCTGCGGCCGCTTTATTCATCAAATAATTTTG
HS52	5' homology arm F for recombination R317 (HA tag)	CTTTTGCAAAAAGCTTCTTCCAAAATCAATGGTGAAAAAA
HS53	5' homology arm R for recombination R317 (HA tag)	AACATCGTATGGGTAGTGATTGGTTTTTGAAATAATTCTG
HS54	3' homology arm F for recombination R317 (HA tag)	AATAGTCCTTTAGAATTATCAACAAGTTATCAATTAATT

HS55	3' homology arm R for recombination R317 (HA tag)	CTTATCGCTGCGGCCGCATCCAAACAAAAAATACATGGG
HS56	5' homology arm F for recombination L446 (HA tag)	CTTTTGCAAAAAGCTTGACGTAATATATTATGACCAC
HS57	5' homology arm R for recombination L446 (HA tag)	AACATCGTATGGGTATTTATTGTCAAATTTATTTTTAAT
HS58	3' homology arm F for recombination L446 (HA tag)	AATAGTCCTTTAGACAAAATAATTGTATTTGACTATT
HS59	3' homology arm R for recombination L446 (HA tag)	CTTATCGCTGCGGCCGCCTATGTTGATGGACATAAAGT
HS60	5' homology arm F for recombination L593 (HA tag)	CTTTTGCAAAAAGCTTCAACAAATGTGGATATCTAAAAAT
HS61	5' homology arm R for recombination L593 (HA tag)	AACATCGTATGGGTAGGAAAATTTTCGTTCTCTAAACC
HS62	3' homology arm F for recombination L593 (HA tag)	AATAGTCCTTTAGAAATTTTTTTAACACAAAATTCATT
HS63	3' homology arm R for recombination L593 (HA tag)	CTTATCGCTGCGGCCGCAAAAGAACTCCAAGAAAAAAACT
HS64	5' homology arm F for recombination R595 (HA tag)	CTTTTGCAAAAAGCTTATCTTCATTATAATCCAAACAAT
HS65	5' homology arm R for recombination R595 (HA tag)	AACATCGTATGGGTACCAATCGTTAAATAAATCAGCCA
HS66	3' homology arm F for recombination R595 (HA tag)	AATAGTCCTTTAGAATATTAAATGTGTAAAAAAAAATTG
HS67	3' homology arm R for recombination R595 (HA tag)	CTTATCGCTGCGGCCGCTATTGAGTTATACTTAATACC
HB831	5' homology arm F for recombination L410 (HA tag)	CTTTTGCAAAAAGCTTCCGATAGACTTGTATTGGATCGCG
HB832	5' homology arm R for recombination L410 (HA tag)	GAACATCGTATGGGTAGAATAAAGAAAGGGGTTGGTTAGG
HB833	3' homology arm F for recombination L410 (HA tag)	AAATAGTCCTTTAGAAATAATAGTAAAGTCAGGCTAAAAT
HB834	3' homology arm R for recombination L410 (HA tag)	CTTATCGCTGCGGCCGCGTTATGAAGTCAGTATATATGCAAA
HB835	5' homology arm F for recombination L454 (HA tag)	CTTTTGCAAAAAGCTTGGGCCCGGTAGAGGATATGGATATCTTACT
HB836	5' homology arm R for recombination L454 (HA tag)	AACATCGTATGGGTATATGTTGTATTTGATATTAAAAGGA

HB837	3' homology arm F for recombination L454 (HA tag)	AAATAGTCCTTTAGAAATATGCATAATACTGTAAATGTT
HB838	3' homology arm R for recombination L454 (HA tag)	CTTATCGCTGCGGCCGCGGTTATGTAGAATGTAAATACCAAC
HB839	5' homology arm F for recombination L264 (HA tag)	CTTTTGCAAAAAGCTTCGGCATTCACTGTTAAATATG
HB840	5' homology arm R for recombination L264 (HA tag)	AACATCGTATGGGTATGGATAAATAAATAATATATTATTG
HB841	3' homology arm F for recombination L264 (HA tag)	AAATAGTCCTTTAGAACATTATTAAGTTATTTATCTAAAT
HB842	3' homology arm R for recombination L264 (HA tag)	CTTATCGCTGCGGCCGCCATTGTATTCAATATATCATTGTC
HB843	5' homology arm F for recombination R721 (HA tag)	CTTTTGCAAAAAGCTTCAGGCTCTGATGAATCACTCGA
HB844	5' homology arm R for recombination R721 (HA tag)	AACATCGTATGGGTATTGAGAATCATCAATTGATATTGGT
HB845	3' homology arm F for recombination R721 (HA tag)	AAATAGTCCTTTAGATTTTTCAAATCCCAATTTTTTGCA
HB846	3' homology arm R for recombination R721 (HA tag)	CTTATCGCTGCGGCCGCCTCTGGGCAAAAGTATGTGAAAC
HB1044	5' homology arm F for recombination L274 (HA tag)	CTTTTGCAAAAAGCTTGAACCTTATTCATTCGAAGGAATGA
HB1045	5' homology arm R for recombination L274 (HA tag)	AACATCGTATGGGTAGGGGAGAGGTTCAAGTTGATCTTG
HB1046	3' homology arm F for recombination L274 (HA tag)	AAATAGTCCTTTAGAATTTCTAGACATTTTAACCCC
HB1047	3' homology arm R for recombination L274 (HA tag)	CTTATCGCTGCGGCCGCCCACCAATTCATGGTTTTTCGAAGG
HS68	5' homology arm F for recombination R443 (KO)	CTTTTGCAAAAAGCTTTTATACTCGCCAACACCTCCTC
HS69	5' homology arm R for recombination R443 (KO)	AATTGCTAATATTTTGGTACTTGATAAGTTGGACGAACTG
HS70	3' homology arm F for recombination R443 (KO)	AATAGTCCTTTAGACTCCGGACAAAAATCTTGAAT
HS71	3' homology arm R for recombination R443 (KO)	CTTATCGCTGCGGCCGCCACCTAGATTTGATGAATTAGAAT
HB1200	Cis complementation R443	CTTTTGCAAAAAGCTTGGTTGAGTGGGTTGAGCA
HB1201	Cis complementation R443	GAACATCGTATGGGTATTGATTCATGTTAGATCGGATGAA

HB1202	Mutation for cis complementation R443 (nucleophilic cys replaced by ser)	TCCGAGTTCCCCACACTGTAAAAATTTCTC
HB1203	Mutation for cis complementation R443 (nucleophilic cys replaced by ser)	TGTGGGGAACTCGGATTATTAAAATAGTATAA
HB1204	Mutation for cis complementation R443 (resolving cys replaced by ser)	CCCACACTCTAAAAATTTCTCGTCAACATGGGA
HB1205	Mutation for cis complementation R443 (resolving cys replaced by ser)	TTTTTAGAGTGTGGGCAACTCGGAT
HS72	Genotyping R443 KO	CTCACGAGAAATGGTGTTAAC
HS73	Genotyping R443 KO	CTGATACAGTAGGTTCGGTCG
HS74	Genotyping R443	TGCAAGTTATTCAGCCTCATC
HS75	Genotyping R443	CGAACAATCTAGACCAAATGAG
HS76	Genotyping L515	CTTTACGATCAACAACATCAAAC
HS77	Genotyping L515	CTAATGTATTGTGCCATCCAA
HS78	Genotyping L330	CATGAATCAGATCCCTATTCAG
HS79	Genotyping L330	GATACCGAAAGATCACGTCTC
HS80	Genotyping R710	GCATTCGAGAATTCACCAGC
HS81	Genotyping R710	GGGGCCCCTATTGAAAATGA
HS82	Genotyping R387	CCTCTTTGAATCCATATCTTCC
HS83	Genotyping R387	CCAGACGATGATTTTGGCAG
HS84	Genotyping L274	GGTTGGTTCCAATGTCAAGCTAC
HS85	Genotyping L274	GATCATGTTAGATTTCCAACCTG
HS86	Genotyping R347	GCTTCCTTACTATTCCCTTTG
HS87	Genotyping R347	GATACTTATTGTTGTCGCTAC
HS88	Genotyping R287	GGATATCAAACTACAACGACAAAC
HS89	Genotyping R287	GCAACATCAAATCCCGGAGC
HS90	Genotyping R513b	CAATGGGACTAACTACAGGCG

HS91	Genotyping R513b	CCTCATCATCATCAGATGCAC
HS92	Genotyping L567	GTTGTCAATGGTCTTATTGATC
HS93	Genotyping L567	GCCAAAAAAGTCAAAATGCATG
HS94	Genotyping L487	GTTTCCAAACTCGAACAAGATG
HS95	Genotyping L487	CCTTCTGGTGGAAACCATCC
HS96	Genotyping L323	GCATGTCAGTTTAGAGTTGTCC
HS97	Genotyping L323	CCATATCAATCCCAAAAACTTC
HS98	Genotyping R335	CAATCAATATAATCAACAACCAAATC
HS99	Genotyping R335	AAACATACCAATCTCAATAATCG
HS100	Genotyping R317	GCTGATTTGTATCTCAATGTCG
HS101	Genotyping R317	GACCTTGAACCGTATTTTAATCTC
HS102	Genotyping L446	CTGGAATACATTTTACAGTAGTAGG
HS103	Genotyping L446	GGATCGTCTTTACCAAAAATATCC
HS104	Genotyping L593	GCAATTTGCCAATGGAAAAC
HS105	Genotyping L593	GAGTTCAGATAAAAAGAGTTCAG
HS106	Genotyping R595	CAACAGGATCTTTCGACAGACG
HS107	Genotyping R595	GAATTGGGCGCAATCTCTAC
HB918	Genotyping L410	GTTTCGAACGTCTCAATG
HB919	Genotyping L410	GCTGTATCAAATATTGGAACCG
HB932	Genotyping L454	GGACAAGCAAATCATGATGG
HB933	Genotyping L454	GTCGATCGAAAGAAACTTGCC
HB934	Genotyping L264	GTACAACACCATTTACTCTCG
HB935	Genotyping L264	TGACATTGTTTTTGTTGTACGG
HB928	Genotyping R721	GGACTTGAATTAGATTTTAGTCCAG
HB929	Genotyping R721	CAAACAATATCATGAGTTCGAG
HB1138	Genotyping L274	CCCAAGAAACTAGCTGATAC
HB1139	Genotyping L274	GATTTCCAACCTGATTCAACAC

HB170	Check integration of 3'homology arm in the plasmid (vAS1)	ATGCCCTGCCCCTAATAATATTATTTTTAAATAATCAAAT
HB270	Check integration of 5'homology arm in the plasmid (vAS1)	AAATAAGAACAAGAGttaAGCGTAATCTGGAACGTCATATGG
HS108 (c)	Nourseothricin cassette selection for Genotyping	GTGTCGTCAAGAGTGGTAC
HB412 (d)	Geneticin (neomycin) cassette selection for Genotyping	CTTCTTGACGAGTTCTTCTGA
HB413 (e)	Geneticin (neomycin) cassette selection for Genotyping	GAACAAGATGGATTGCACGCA
CG69	qPCR NAT selection	TACCACTCTTGACGACACGG
CG70	qPCR NAT selection	AGTACGAGACGACCACGAAG
CG71	qPCR mimi genome	TCCTAAACCTCTTCAAGGAGAC
CG72	qPCR mimi genome	TACCGAACATTGACGCGAC
HB223	swap NAT for NeoR	TAATATTATTTTTAAATAATC
HB224	swap NAT for NeoR	GATATCGGTGGCTCTAGC
HB225	swap NAT for NeoR	AGAGCCACCGATATCATGATTGAACAAGATGGATTGCACG
HB226	swap NAT for NeoR	TTAAAAATAATATTATCAGAAGAACTCGTCAAGAAGGC
