# Supplementary material for: Elucidating the protein interaction network of one of the largest icosahedral capsids in the virosphere
Source: EMBO J. 2026 Apr 10;45(10):3514–39. doi: 10.1038/s44318-026-00770-8 (PMC13186993; doi:10.1038/s44318-026-00770-8)
Supplement: Supplementary file 4 — Appendix [file 44318_2026_770_MOESM4_ESM.pdf]

Appendix for: Elucidating the protein interaction network of one of the largest icosahedral capsids in the virosphere

Table of Contents

Appendix Supplementary Figures 2

Appendix Figure S1. Improvement of the clustering by extending the dataset beyond mimivirus Taxon 3

Appendix Figure S2. Western blot of endogenously tagged mimivirus proteins 4

Appendix Figure S3. Conservation analysis of the co-IP network 6

Appendix Figure S4. Icosahedral shape enrichment in different virion regions 7

Appendix Figure S5. R443 transmembrane domain and active site 9

Appendix Figure S6. Cysteine mutagenesis and substrate trapping of R443 10

Appendix Figure S7. Fluorescence observation of mimivirus core release during early infection 11

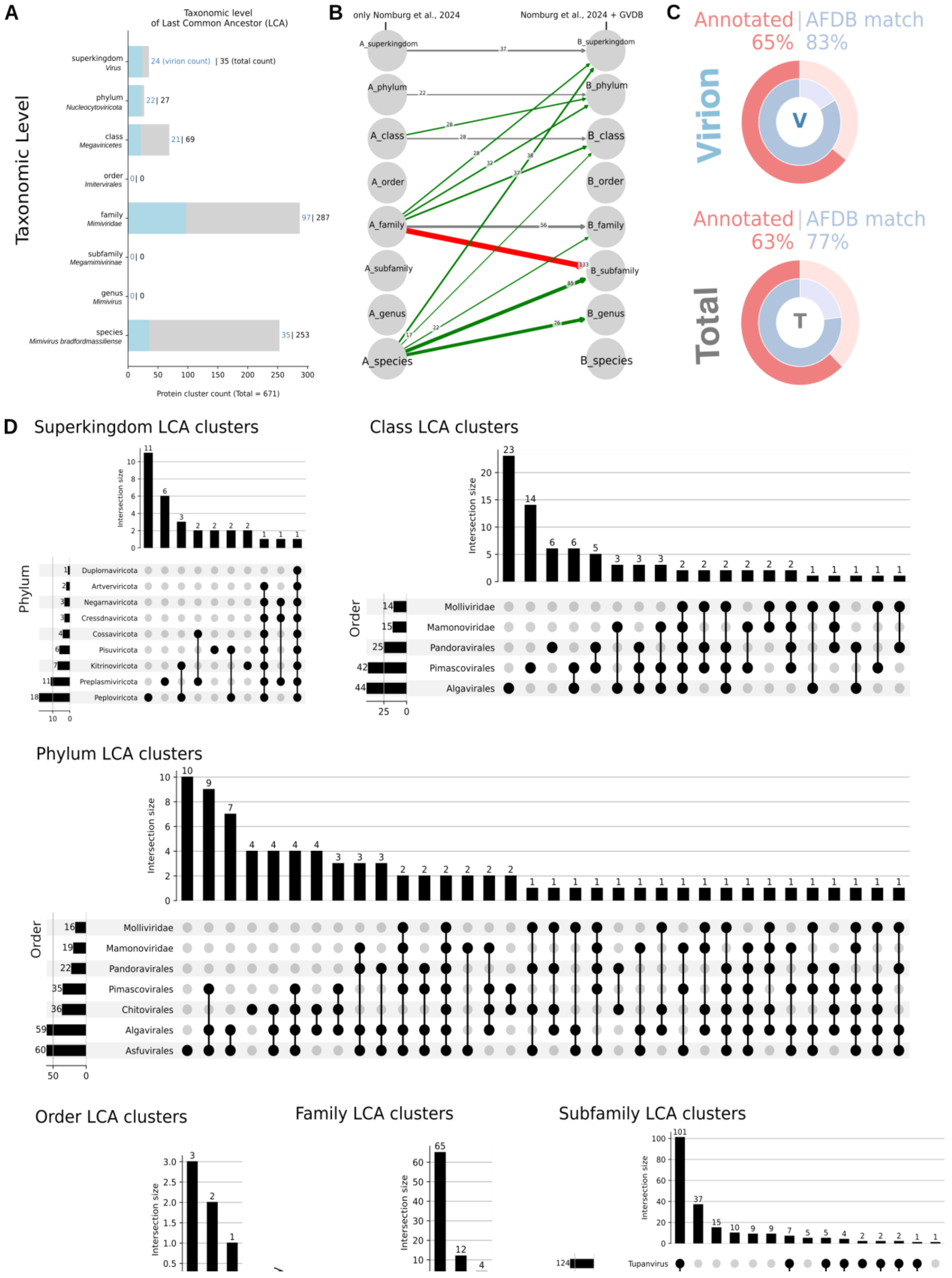
A
Taxonomic level
of Last Common Ancestor (LCA)
Taxonomic Level
superkingdom
Virus
24 (virion count) | 35 (total count)
phylum
Nucleocytoviricota
22| 27
class
Megaviricetes
21| 69
order
Imitervirales
0| 0
family
Mimiviridae
97| 287
subfamily
Megamimivirinae
0| 0
genus
Mimivirus
0| 0
species
Mimivirus bradfordmassiliense
35| 253
Protein cluster count (Total = 671)
B
only Nomburg et al., 2024
Nomburg et al., 2024 + GVDB
A_superkingdom
A_phylum
A_class
A_order
A_family
A_subfamily
A_genus
A_species
B_superkingdom
B_phylum
B_class
B_order
B_family
B_subfamily
B_genus
B_species
C
Annotated | AFDB match
65% 83%
Virion
V
Annotated | AFDB match
63% 77%
Total
T
D
Superkingdom LCA clusters
Intersection size
Phylum
Duplornaviricota
Artverviricota
Negarnaviricota
Cressdnaviricota
Cossaviricota
Pisuviricota
Kitrinoviricota
Preplasmiviricota
Peploviricota
Class LCA clusters
Intersection size
Order
Molliviridae
Mamonoviridae
Pandoravirales
Pimascovirales
Algavirales
Phylum LCA clusters
Intersection size
Order
Molliviridae
Mamonoviridae
Pandoravirales
Pimascovirales
Chitovirales
Algavirales
Asfuvirales
Order LCA clusters
Intersection size
Family LCA clusters
Intersection size
Subfamily LCA clusters
Intersection size
Tupanvirus

Appendix Figure S1. Improvement of the clustering by extending the dataset beyond mimivirus Taxon.

(A) Taxonomic Level of the Last Common Ancestor of the 671 mimivirus clusters (Nomburg et al. (Nomburg et al, 2024) clustering data). (B) Correction of the LCA taxonomy levels obtained after adding 62 giant viruses mostly *Imitervirales*, *Pimascovirales*, *Pandovirales* and some *Asfuvirales*. Taxonomy levels shifts towards the top (resp. bottom) are depicted with green arrows (resp. red arrows). (C) Pie charts indicating the fraction of mimivirus proteins in clusters with AFDB matches (blue), and with PFAM annotations (red) or annotated AFDB homolog, (top) for virion proteins subset, (bottom) total subset. (D) Upset plots showing the sub-level composition of each taxonomy level LCA.

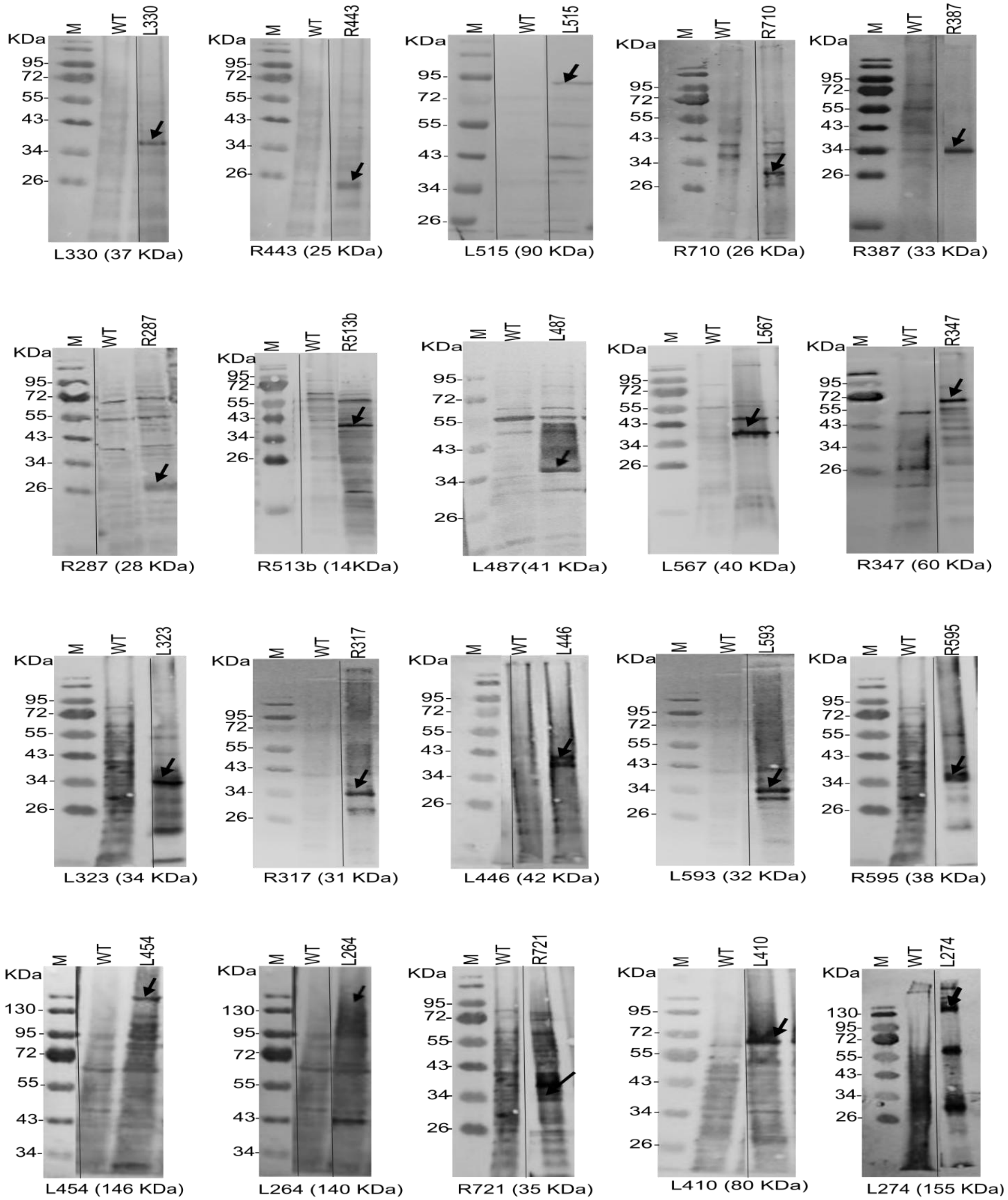

Appendix Figure S2. Western blot of endogenously tagged mimivirus proteins.

Western blot (WB) analyses were performed using anti-HA antibodies to detect the tagged mimivirus proteins (protein bands indicated by black arrows). Acanthamoeba cells were infected with mimivirus at MOI = 10 and collected 6 hpi. Wild-type mimivirus infected cells were included as negative controls. M: molecular weight marker. Control lanes corresponding to proteins run on the same gels were re-

used in the corresponding panels. WB1 included WT, L443 and L330; WB2 included WT, L323, R595 and R721; WB3 included WT, R317 and L593; WB4 included WT, L264 and L454. For each blot, the WT control lane runs alongside the corresponding HA-tagged proteins as displayed in the assembled panels (marker + WT + HA-gene).

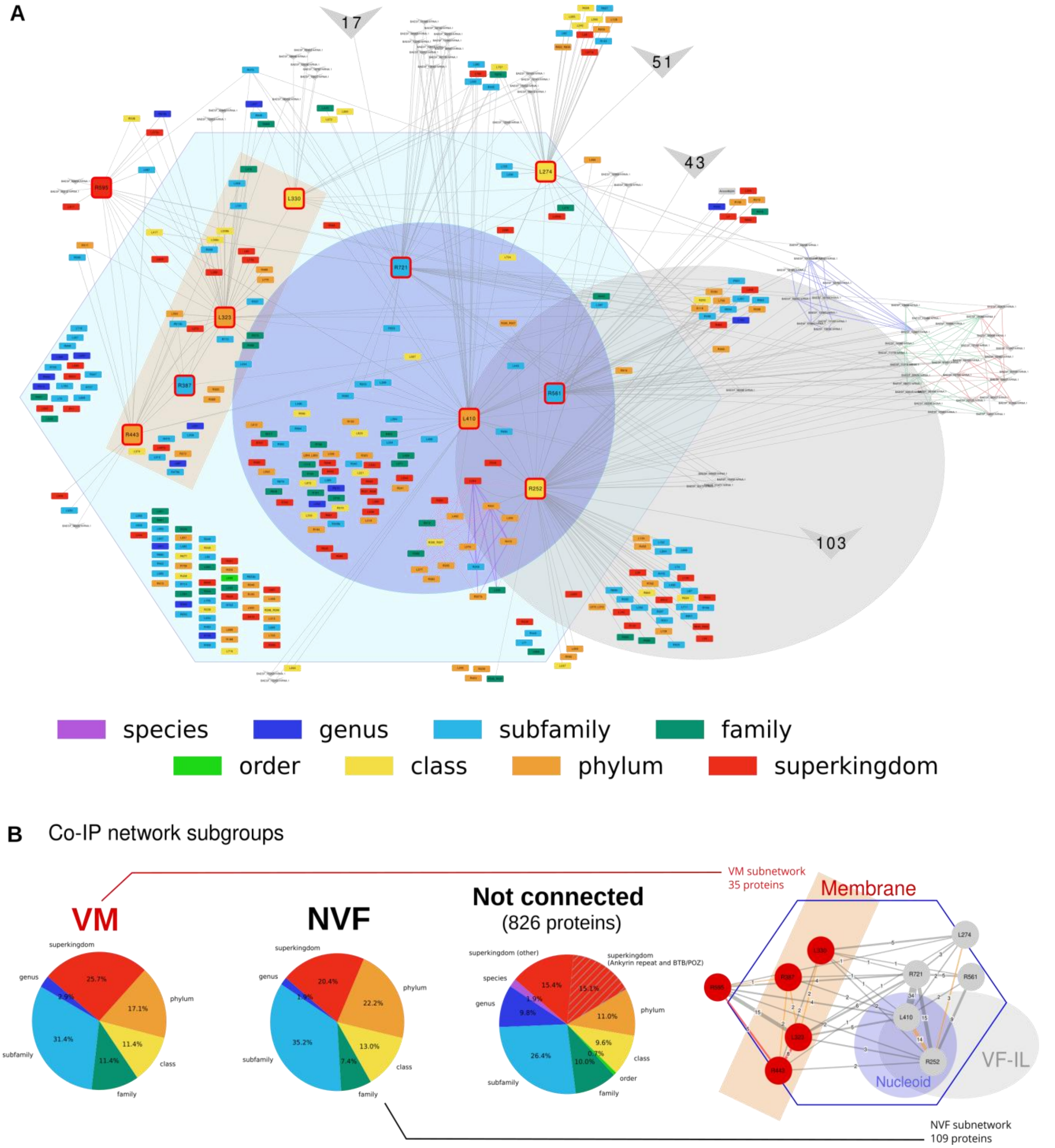

Appendix Figure S3. Conservation analysis of the co-IP network.

(A) Detailed network showing all connections of the co-IP baits with their connected proteins, as well as direct PPI networks as described in Fig. 3. Taxonomy level of the Last Common ancestor (LCA) is shown as node colors from violet (species) to red (superkingdom). (B) LCA taxonomic level conservation is shown by co-IP subnetworks. Both VM and NVF subnetworks show higher subfamily-level and phylum conservation compared to not-connected proteins.

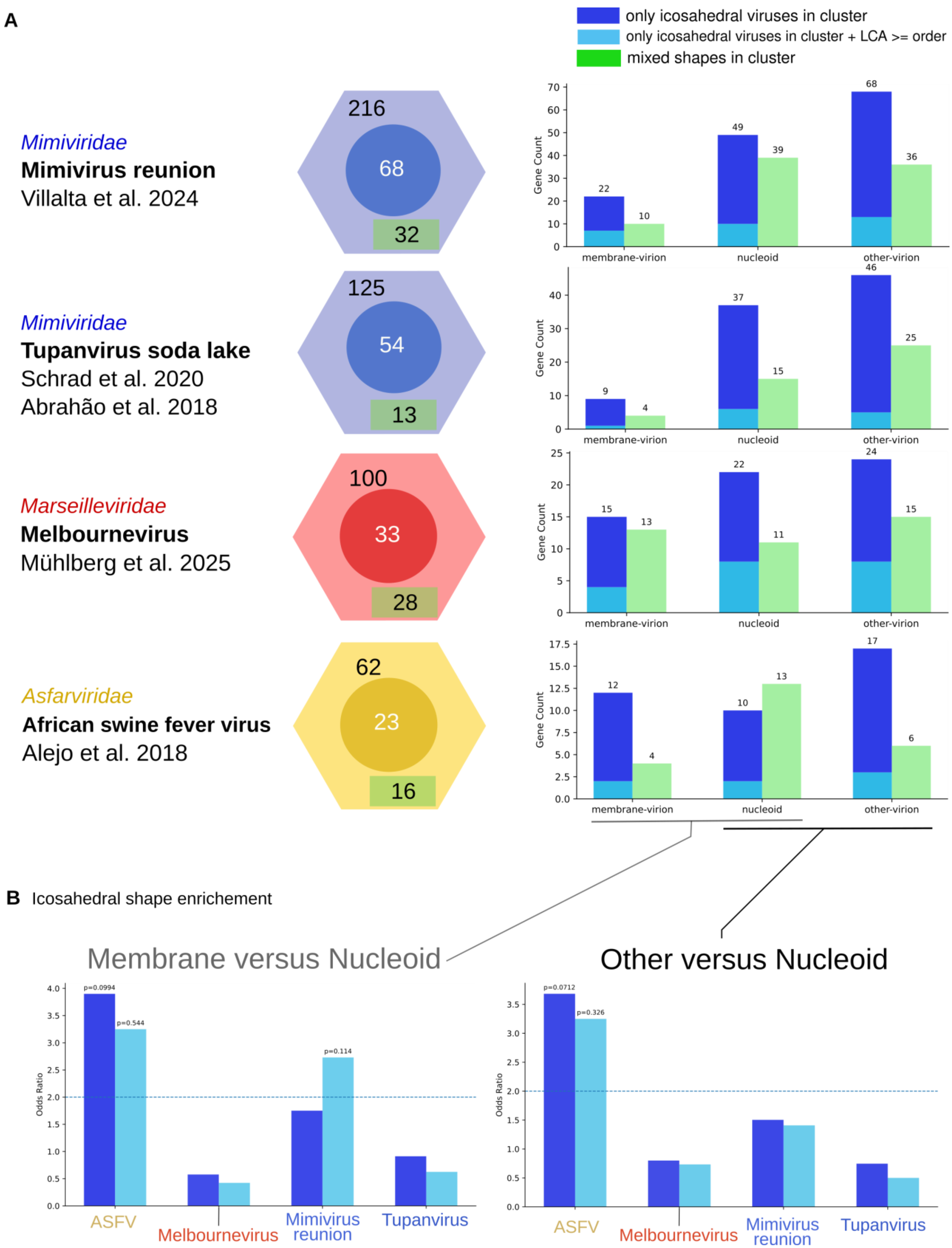
A
only icosahedral viruses in cluster
only icosahedral viruses in cluster + LCA >= order
mixed shapes in cluster
Mimiviridae
Mimivirus reunion
Villalta et al. 2024
216
68
32
Mimiviridae
Tupanvirus soda lake
Schrad et al. 2020
Abrahão et al. 2018
125
54
13
Marseilleviridae
Melbournevirus
Mühlberg et al. 2025
100
33
28
Asfarviridae
African swine fever virus
Alejo et al. 2018
62
23
16
Gene Count
membrane-virion
nucleoid
other-virion
B Icosahedral shape enrichement
Membrane versus Nucleoid
Other versus Nucleoid
Odds Ratio
p=0.0994
p=0.544
p=0.114
p=0.0712
p=0.326
ASFV
Melbournevirus
Mimivirus reunion
Tupanvirus

Appendix Figure S4. Icosahedral shape enrichment in different virion regions.
(A) Extension of the analysis of statistical association between viral shape and protein composition across different virion regions using additional proteomic datasets for different icosahedral viruses. For each virus, (left) its family and original publication is recapitulated, (center) total proteins count in the virion, proteins count in the nucleoid and count of predicted membrane proteins is summarized, (right) protein count per virion region and virion shape (Icosahedral only in blue, mixed shape in green). (B) Odds ratio and corresponding P-value using Fisher's exact test are summarized for all 4 icosahedral viruses, by comparing the protein content (left) in the membrane region versus the nucleoid, and (right) in the other region versus the nucleoid region.

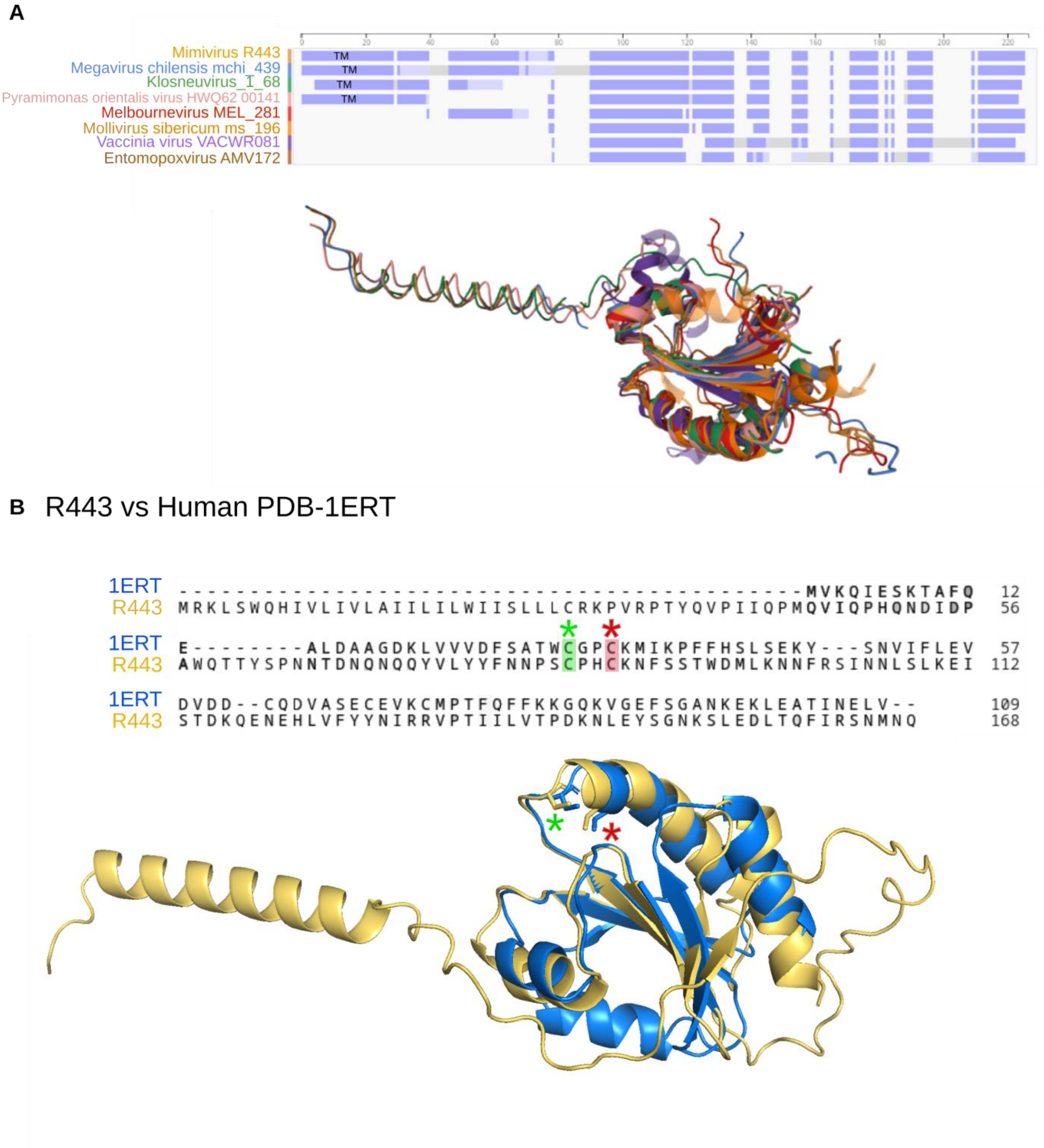

Appendix Figure S5. R443 transmembrane domain and active site.

(A) Structural alignment of selected R443 homologs (Foldmason tool), members of the same cluster with conserved Thioredoxin globular. Transmembrane domain is only conserved in members of the *Imitervirales* order. (B) Alignment of the Human Thioredoxin (RCSB PDB 1ERT) and mimivirus R443. The R443 thioredoxin domain is well conserved and the catalytic CXXC motif is highlighted with green and red stars.

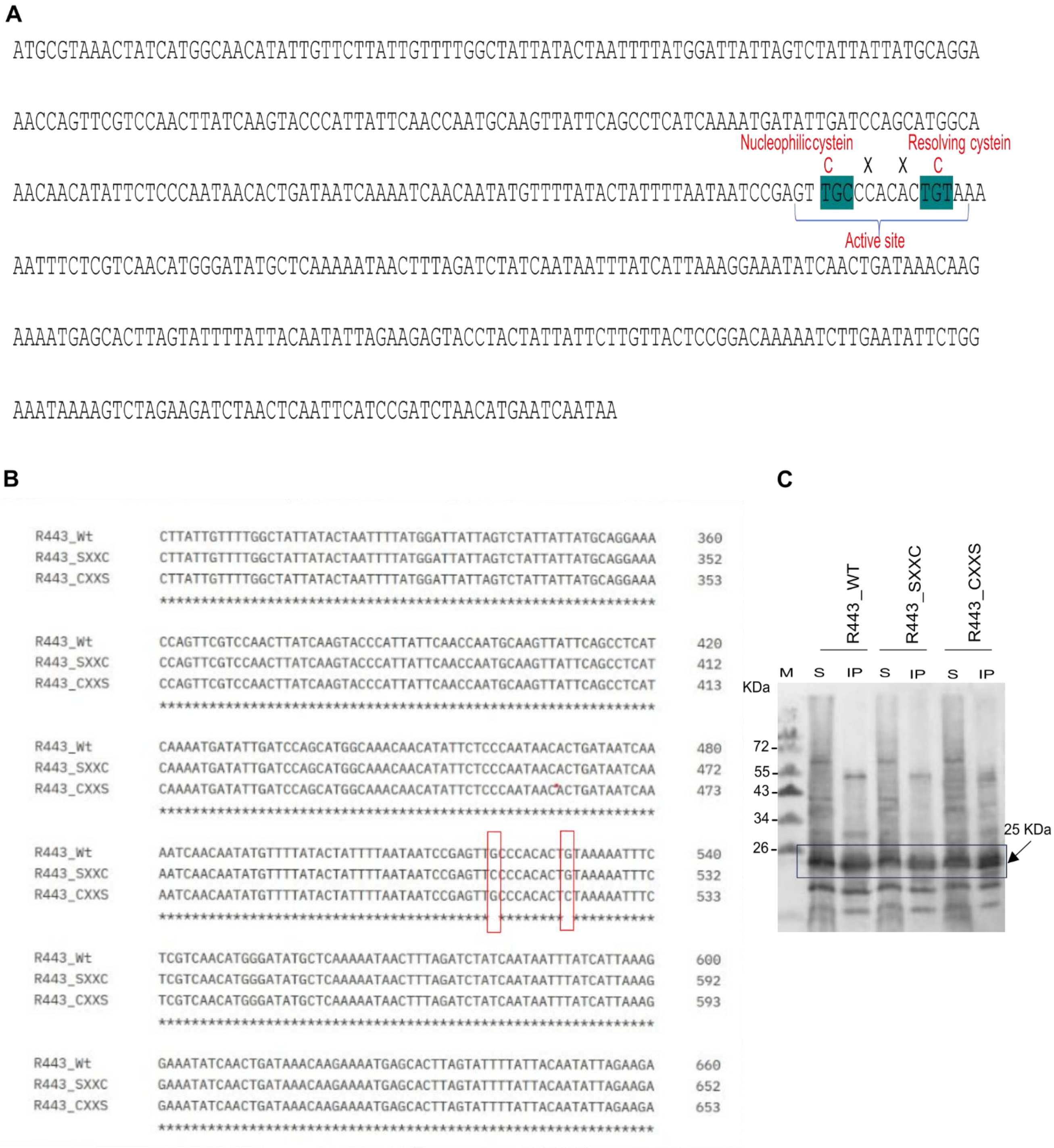

Appendix Figure S6. Cysteine mutagenesis and substrate trapping of R443.

(A) R443 thioredoxin gene sequence and protein active site location. (B) Sequencing confirmation of cysteine mutations. (C) Western blot analysis of R443 substrate-trapping immunoprecipitation using anti-HA antibody. WB is carried out on *A.castellanii* cells at 6hpi (MOI=10). M: marker; S: supernatant; IP: immunoprecipitate.

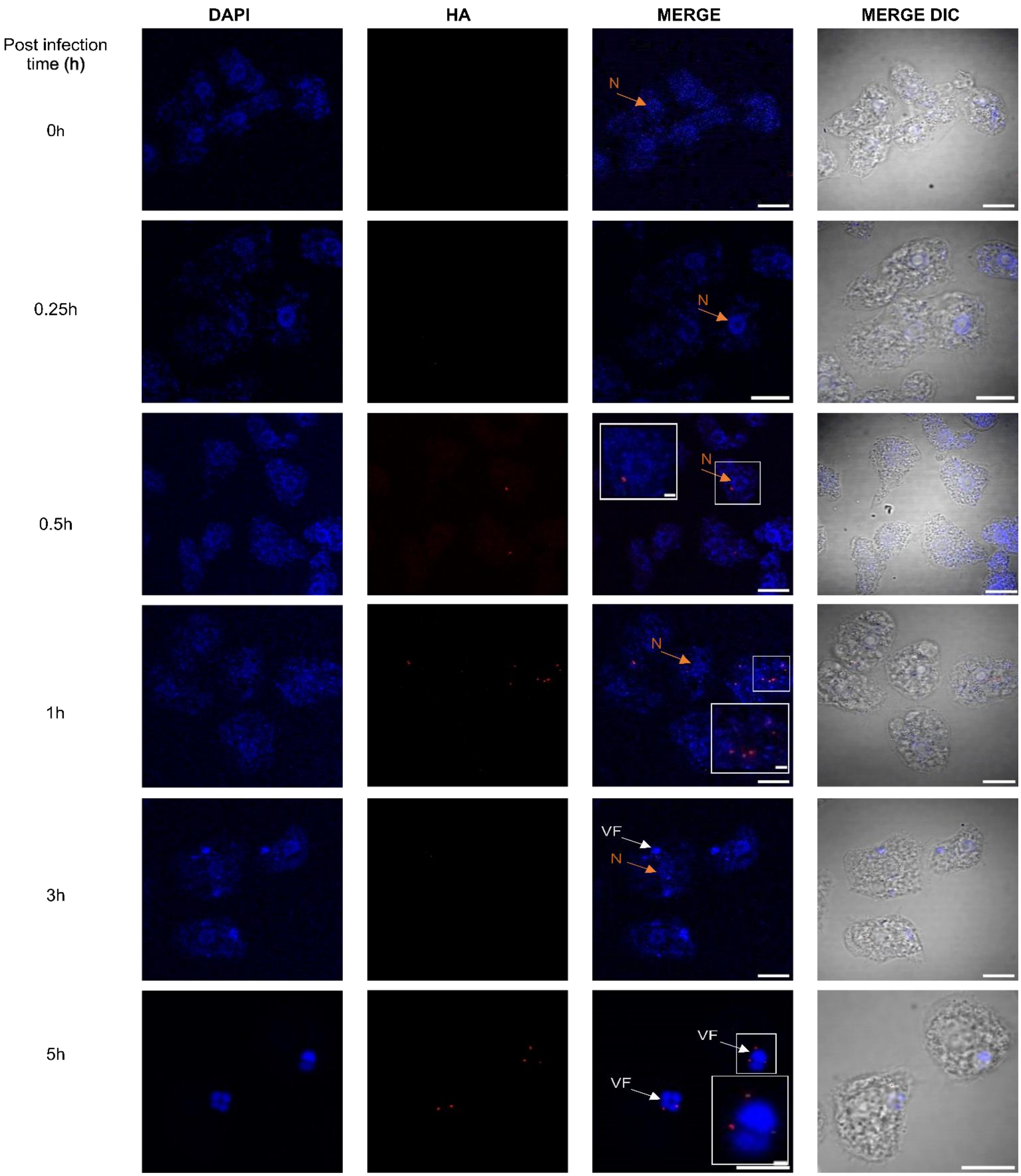

Appendix Figure S7. Fluorescence observation of mimivirus core release during early infection.

Immunofluorescence of L410 major core protein endogenously tagged with 3xHA at the C-terminal at different stages of infection at a MOI=10 (0, 0.25, 0.5,1, 3 and 5 hours). Insets

show higher magnification views of representative puncta. N: nucleus; VF: viral factory. VFs were labelled using DAPI. Scale bar: 10 μm.
