# Supplementary figures and images for: Elucidating the protein interaction network of one of the largest icosahedral capsids in the virosphere (part 1 of 2)
Source: EMBO J. 2026 Apr 10;45(10):3514–39. doi: 10.1038/s44318-026-00770-8 (PMC13186993; doi:10.1038/s44318-026-00770-8)

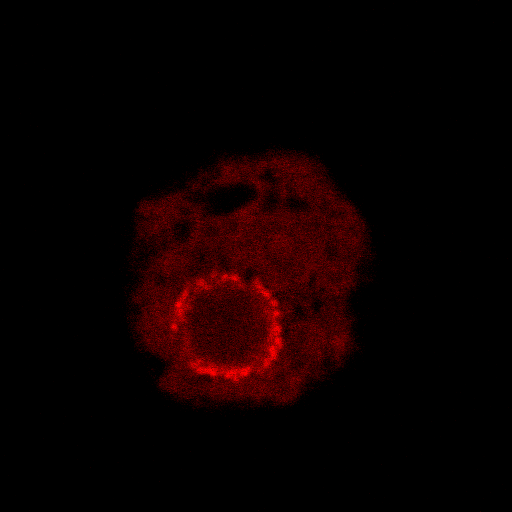

Supplement: Supplementary file 12 — Source data Fig. 2 [file 44318_2026_770_MOESM12_ESM.zip › Figure_2/L264/L246 rfp.tif]

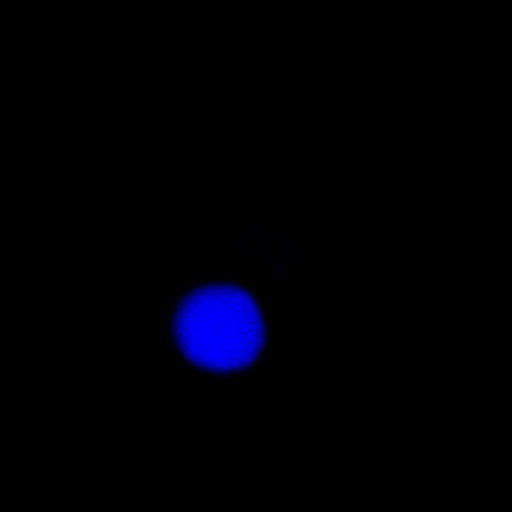

Supplement: Supplementary file 12 — Source data Fig. 2 [file 44318_2026_770_MOESM12_ESM.zip › Figure_2/L264/L264 dapi.tif]

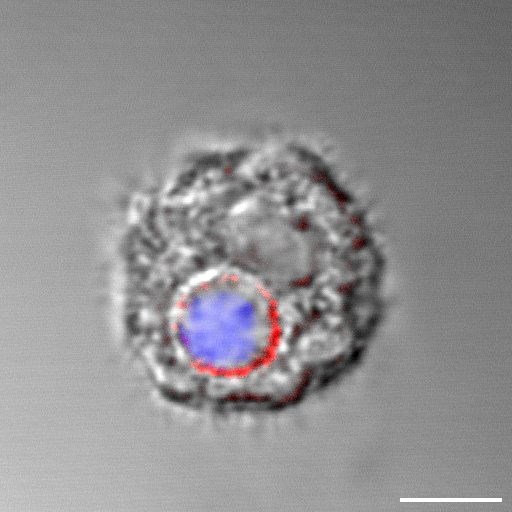

Supplement: Supplementary file 12 — Source data Fig. 2 [file 44318_2026_770_MOESM12_ESM.zip › Figure_2/L264/L264 merge dic.tif]

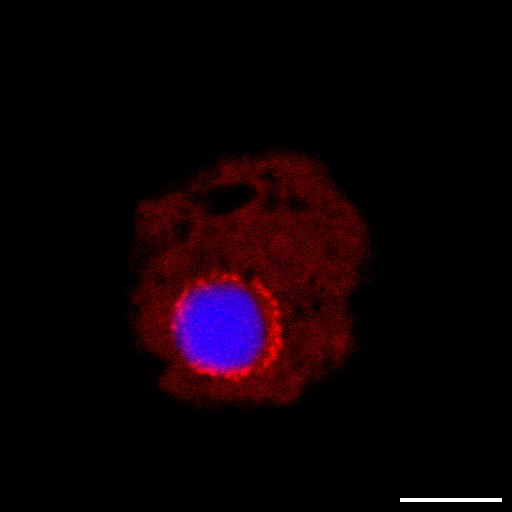

Supplement: Supplementary file 12 — Source data Fig. 2 [file 44318_2026_770_MOESM12_ESM.zip › Figure_2/L264/L264 merge rfp.tif]

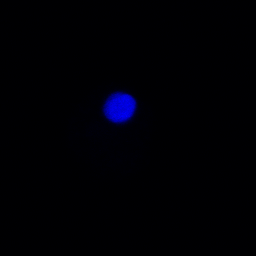

Supplement: Supplementary file 12 — Source data Fig. 2 [file 44318_2026_770_MOESM12_ESM.zip › Figure_2/L274/L274 dapi.tif]

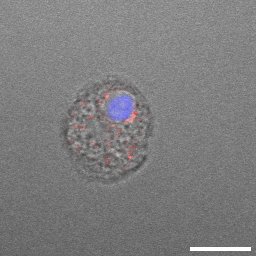

Supplement: Supplementary file 12 — Source data Fig. 2 [file 44318_2026_770_MOESM12_ESM.zip › Figure_2/L274/L274 merge dic.jpg]

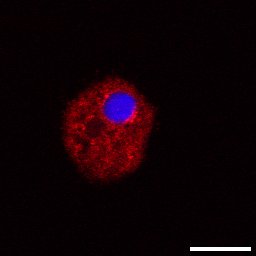

Supplement: Supplementary file 12 — Source data Fig. 2 [file 44318_2026_770_MOESM12_ESM.zip › Figure_2/L274/L274 merge rfp.jpg]

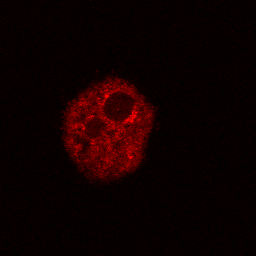

Supplement: Supplementary file 12 — Source data Fig. 2 [file 44318_2026_770_MOESM12_ESM.zip › Figure_2/L274/L274 rfp.tif]

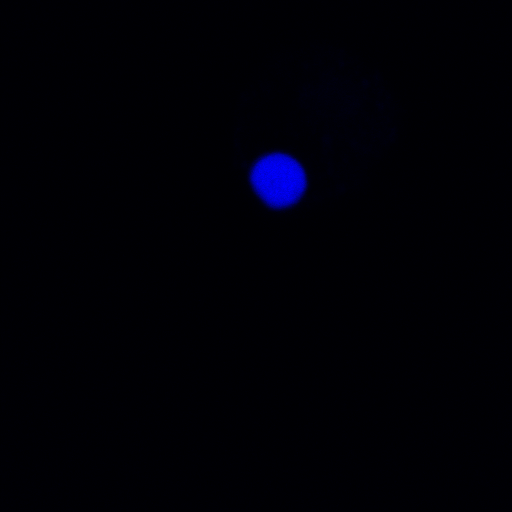

Supplement: Supplementary file 12 — Source data Fig. 2 [file 44318_2026_770_MOESM12_ESM.zip › Figure_2/L323/L323 dapi.tif]

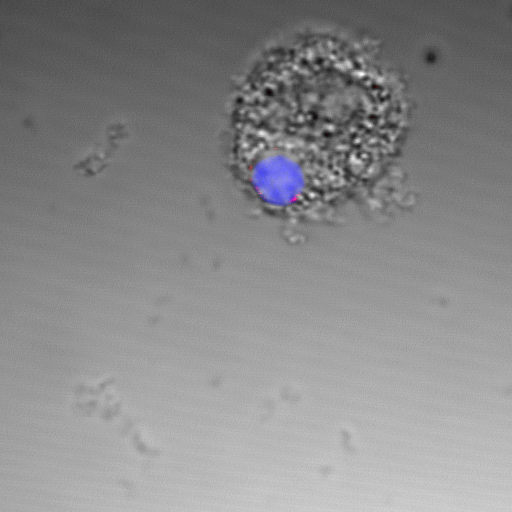

Supplement: Supplementary file 12 — Source data Fig. 2 [file 44318_2026_770_MOESM12_ESM.zip › Figure_2/L323/L323 merge dic.tif]

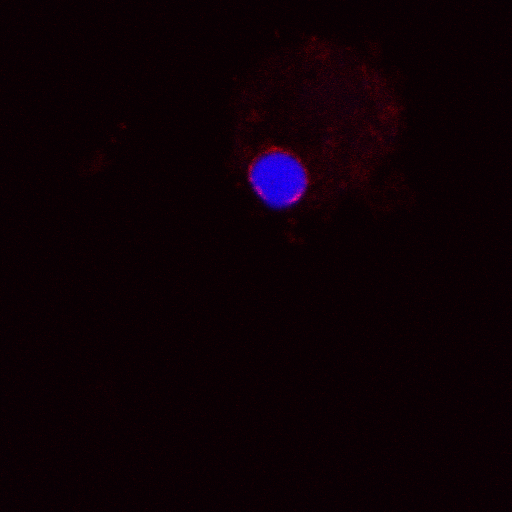

Supplement: Supplementary file 12 — Source data Fig. 2 [file 44318_2026_770_MOESM12_ESM.zip › Figure_2/L323/L323 merge rfp.tif]

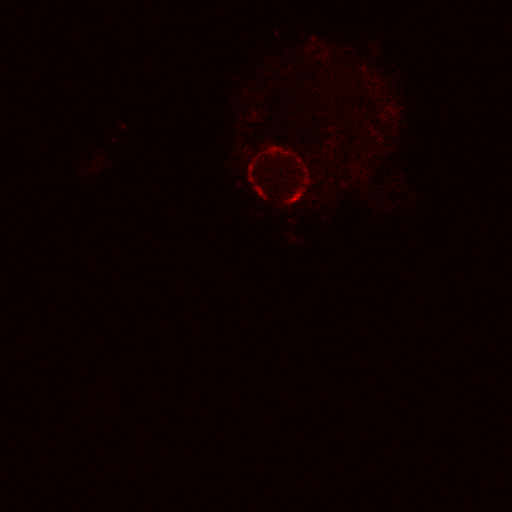

Supplement: Supplementary file 12 — Source data Fig. 2 [file 44318_2026_770_MOESM12_ESM.zip › Figure_2/L323/L323 rfp.tif]

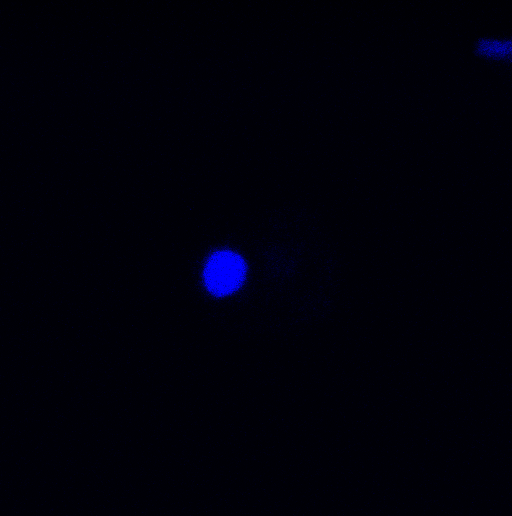

Supplement: Supplementary file 12 — Source data Fig. 2 [file 44318_2026_770_MOESM12_ESM.zip › Figure_2/L330/L330 dapi.tif]

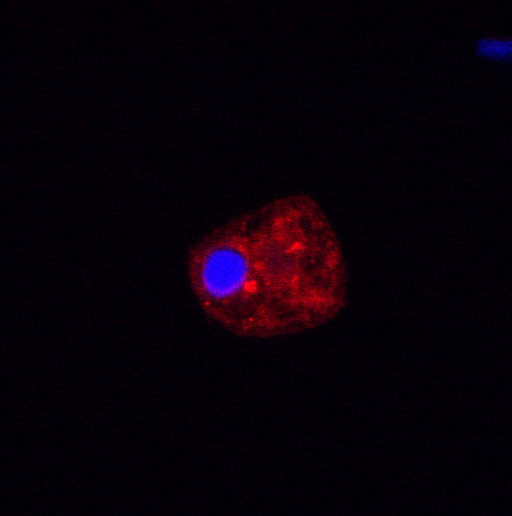

Supplement: Supplementary file 12 — Source data Fig. 2 [file 44318_2026_770_MOESM12_ESM.zip › Figure_2/L330/L330 merge rfp.tif]

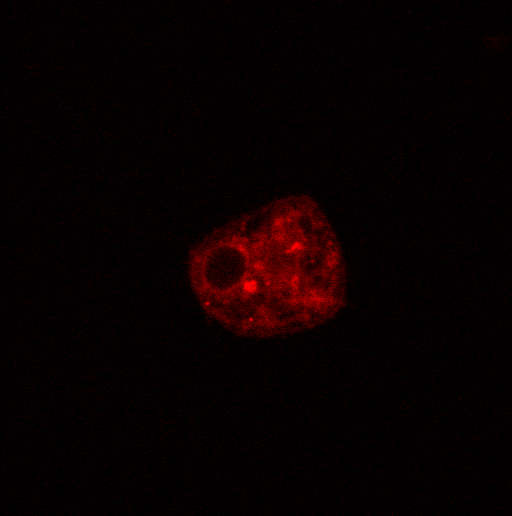

Supplement: Supplementary file 12 — Source data Fig. 2 [file 44318_2026_770_MOESM12_ESM.zip › Figure_2/L330/L330 rfp.tif]

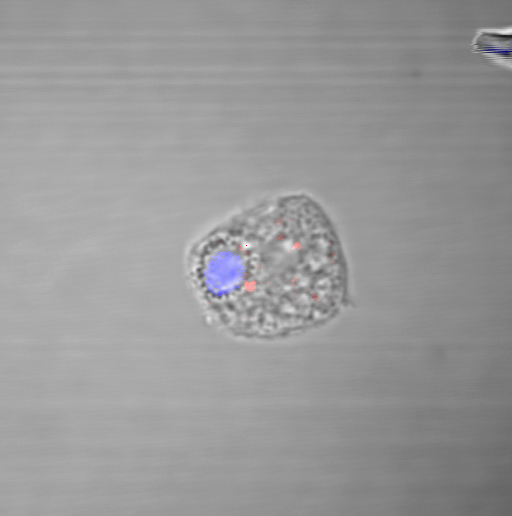

Supplement: Supplementary file 12 — Source data Fig. 2 [file 44318_2026_770_MOESM12_ESM.zip › Figure_2/L330/L33O merge dic.tif]

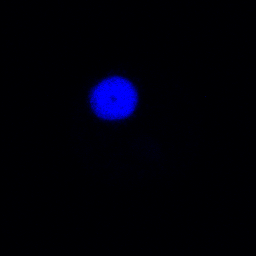

Supplement: Supplementary file 12 — Source data Fig. 2 [file 44318_2026_770_MOESM12_ESM.zip › Figure_2/L410/L410 dapi.tif]

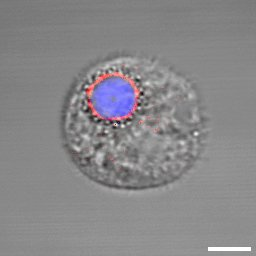

Supplement: Supplementary file 12 — Source data Fig. 2 [file 44318_2026_770_MOESM12_ESM.zip › Figure_2/L410/L410 merge dic.tif]

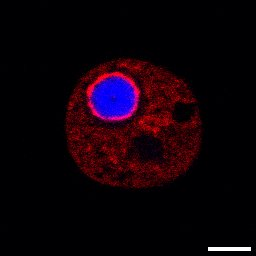

Supplement: Supplementary file 12 — Source data Fig. 2 [file 44318_2026_770_MOESM12_ESM.zip › Figure_2/L410/L410 merge rfp.tif]

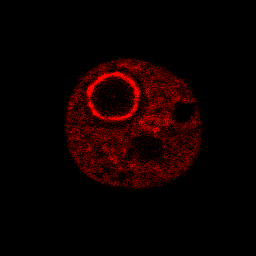

Supplement: Supplementary file 12 — Source data Fig. 2 [file 44318_2026_770_MOESM12_ESM.zip › Figure_2/L410/L410 rfp.tif]

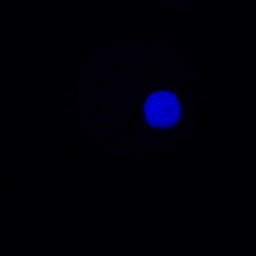

Supplement: Supplementary file 12 — Source data Fig. 2 [file 44318_2026_770_MOESM12_ESM.zip › Figure_2/L446/L446 dapi.tif]

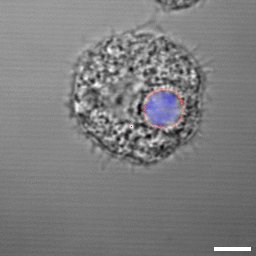

Supplement: Supplementary file 12 — Source data Fig. 2 [file 44318_2026_770_MOESM12_ESM.zip › Figure_2/L446/L446 merge dic.tif]

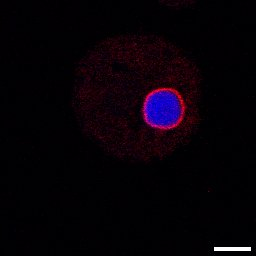

Supplement: Supplementary file 12 — Source data Fig. 2 [file 44318_2026_770_MOESM12_ESM.zip › Figure_2/L446/L446 merge rfp .tif]

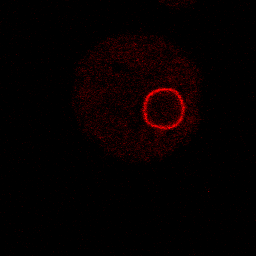

Supplement: Supplementary file 12 — Source data Fig. 2 [file 44318_2026_770_MOESM12_ESM.zip › Figure_2/L446/L446 rfp.tif]

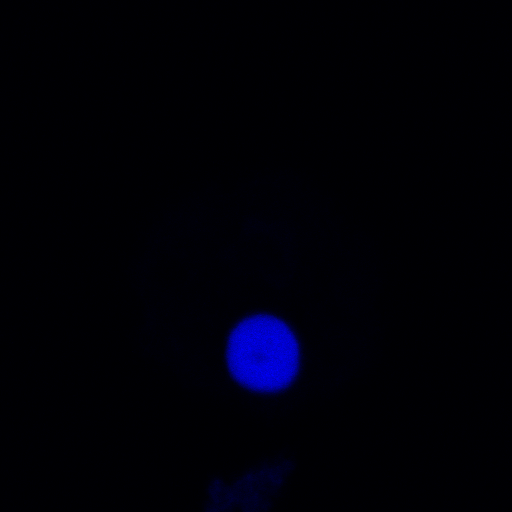

Supplement: Supplementary file 12 — Source data Fig. 2 [file 44318_2026_770_MOESM12_ESM.zip › Figure_2/L454/L454 dapi.tif]

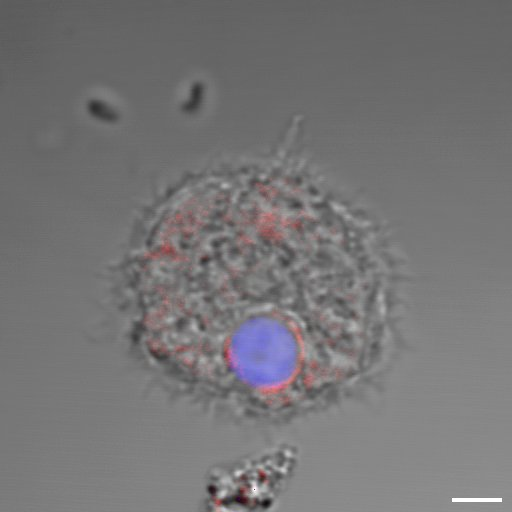

Supplement: Supplementary file 12 — Source data Fig. 2 [file 44318_2026_770_MOESM12_ESM.zip › Figure_2/L454/L454 merge dic.tif]

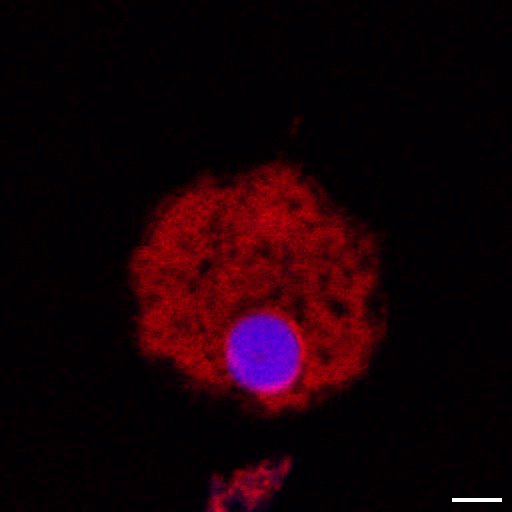

Supplement: Supplementary file 12 — Source data Fig. 2 [file 44318_2026_770_MOESM12_ESM.zip › Figure_2/L454/L454 merge rfp.tif]

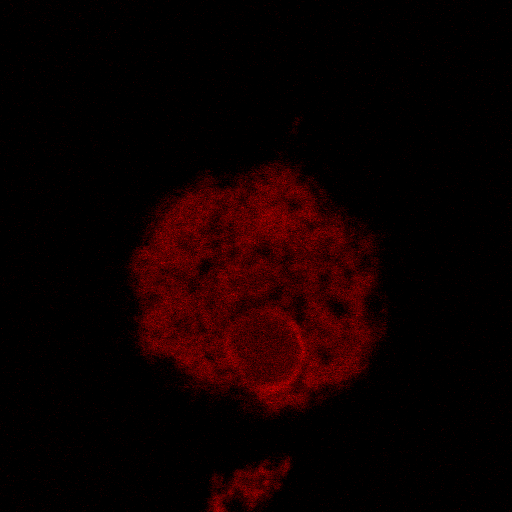

Supplement: Supplementary file 12 — Source data Fig. 2 [file 44318_2026_770_MOESM12_ESM.zip › Figure_2/L454/L545 rfp.tif]

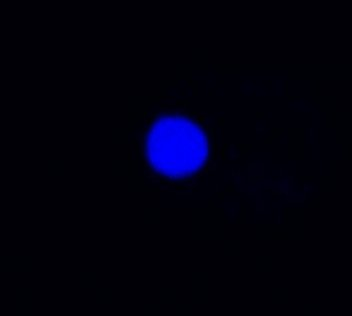

Supplement: Supplementary file 12 — Source data Fig. 2 [file 44318_2026_770_MOESM12_ESM.zip › Figure_2/L487/L487 dapi.tif]

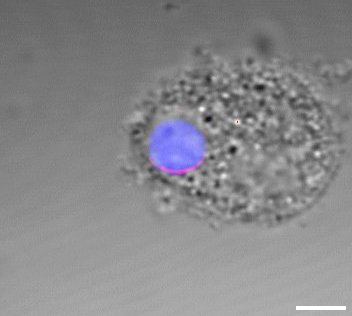

Supplement: Supplementary file 12 — Source data Fig. 2 [file 44318_2026_770_MOESM12_ESM.zip › Figure_2/L487/L487 merge dic.tif]

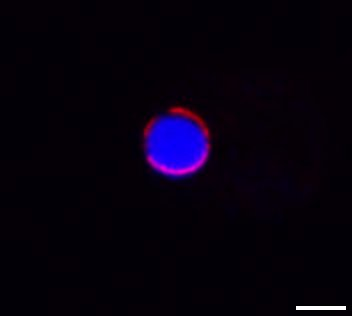

Supplement: Supplementary file 12 — Source data Fig. 2 [file 44318_2026_770_MOESM12_ESM.zip › Figure_2/L487/L487 merge rfp.tif]

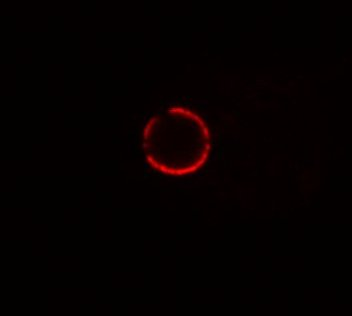

Supplement: Supplementary file 12 — Source data Fig. 2 [file 44318_2026_770_MOESM12_ESM.zip › Figure_2/L487/L487 rfp.tif]

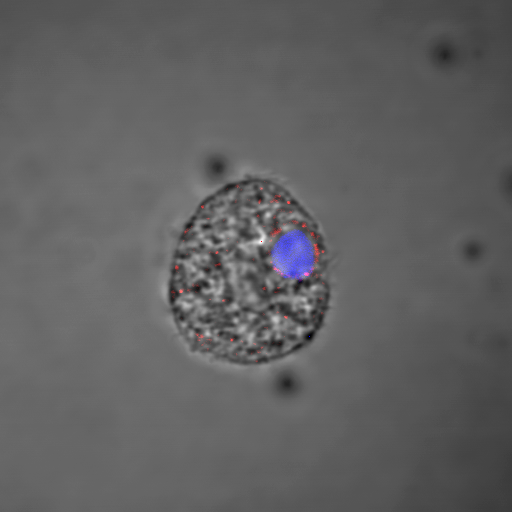

Supplement: Supplementary file 12 — Source data Fig. 2 [file 44318_2026_770_MOESM12_ESM.zip › Figure_2/L515/CL515 merge dic.tif]

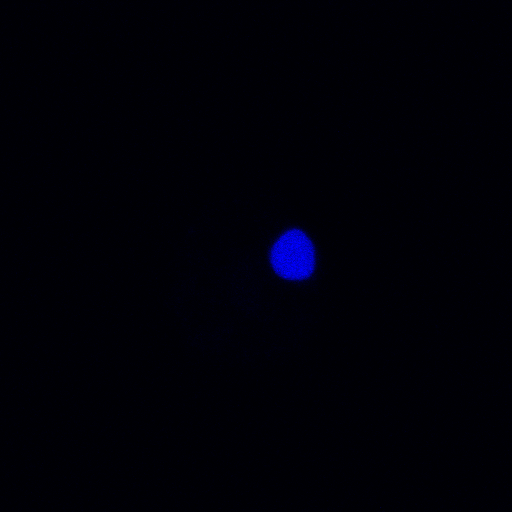

Supplement: Supplementary file 12 — Source data Fig. 2 [file 44318_2026_770_MOESM12_ESM.zip › Figure_2/L515/L515 dapi.tif]

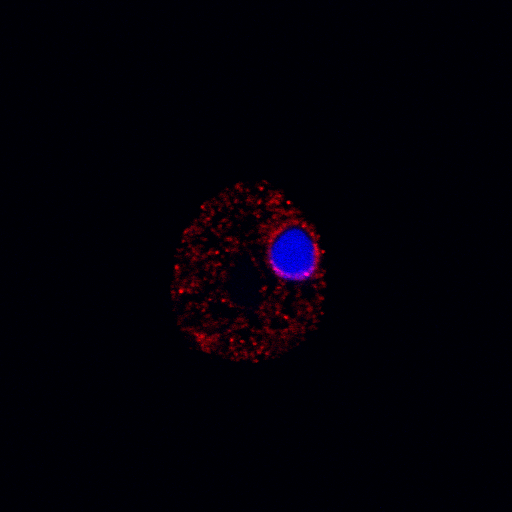

Supplement: Supplementary file 12 — Source data Fig. 2 [file 44318_2026_770_MOESM12_ESM.zip › Figure_2/L515/L515 merge rfp.tif]

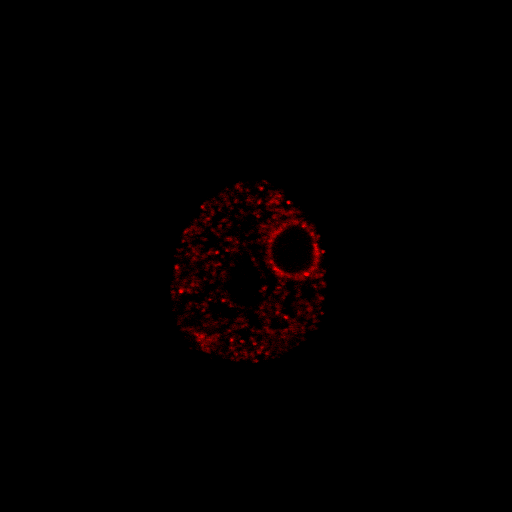

Supplement: Supplementary file 12 — Source data Fig. 2 [file 44318_2026_770_MOESM12_ESM.zip › Figure_2/L515/L515 rfp.tif]

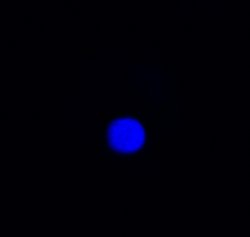

Supplement: Supplementary file 12 — Source data Fig. 2 [file 44318_2026_770_MOESM12_ESM.zip › Figure_2/L567/L567 dapi.tif]

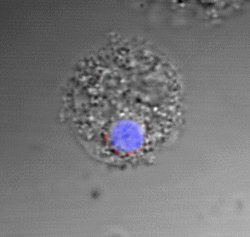

Supplement: Supplementary file 12 — Source data Fig. 2 [file 44318_2026_770_MOESM12_ESM.zip › Figure_2/L567/L567 merge dic.tif]

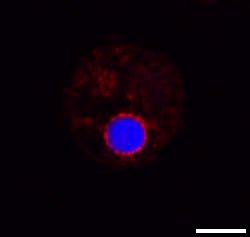

Supplement: Supplementary file 12 — Source data Fig. 2 [file 44318_2026_770_MOESM12_ESM.zip › Figure_2/L567/L567 merge rfp.tif]

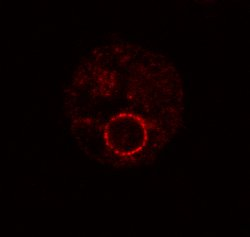

Supplement: Supplementary file 12 — Source data Fig. 2 [file 44318_2026_770_MOESM12_ESM.zip › Figure_2/L567/L567 rfp.tif]

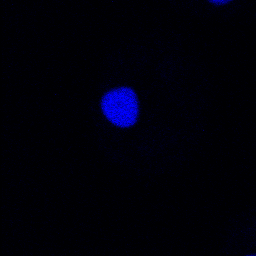

Supplement: Supplementary file 12 — Source data Fig. 2 [file 44318_2026_770_MOESM12_ESM.zip › Figure_2/L593/L593 dapi.tif]

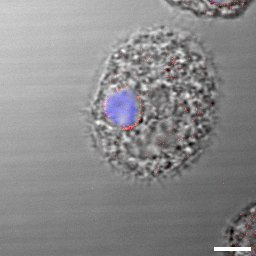

Supplement: Supplementary file 12 — Source data Fig. 2 [file 44318_2026_770_MOESM12_ESM.zip › Figure_2/L593/L593 merge dic .tif]

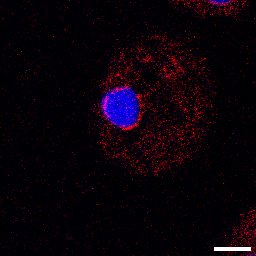

Supplement: Supplementary file 12 — Source data Fig. 2 [file 44318_2026_770_MOESM12_ESM.zip › Figure_2/L593/L593 merge rfp.tif]

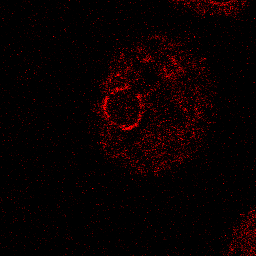

Supplement: Supplementary file 12 — Source data Fig. 2 [file 44318_2026_770_MOESM12_ESM.zip › Figure_2/L593/L593 rfp.tif]

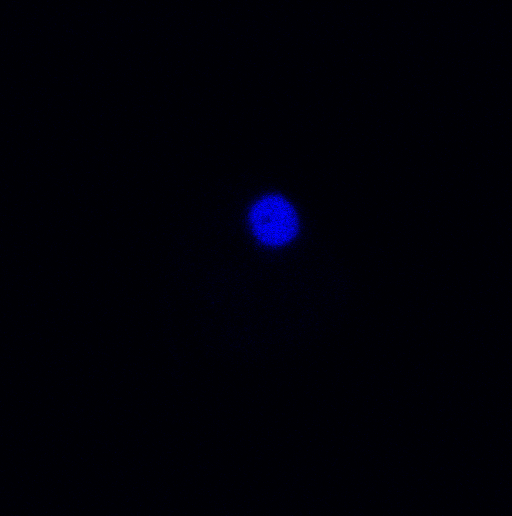

Supplement: Supplementary file 12 — Source data Fig. 2 [file 44318_2026_770_MOESM12_ESM.zip › Figure_2/R287/R287 dapi.tif]

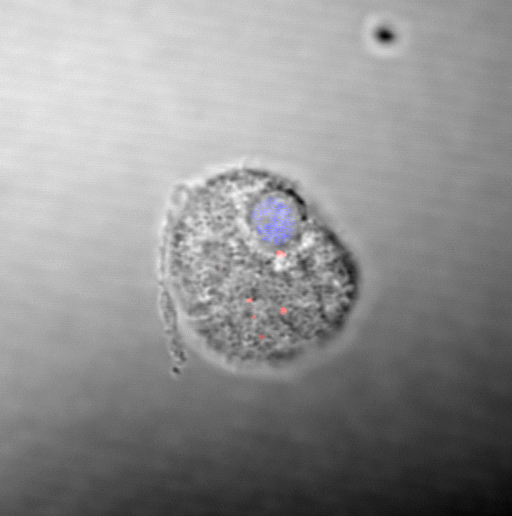

Supplement: Supplementary file 12 — Source data Fig. 2 [file 44318_2026_770_MOESM12_ESM.zip › Figure_2/R287/R287 merge dic.tif]

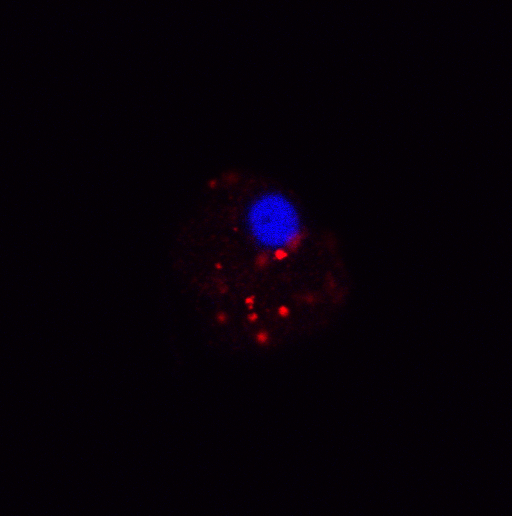

Supplement: Supplementary file 12 — Source data Fig. 2 [file 44318_2026_770_MOESM12_ESM.zip › Figure_2/R287/R287 merge rfp.tif]

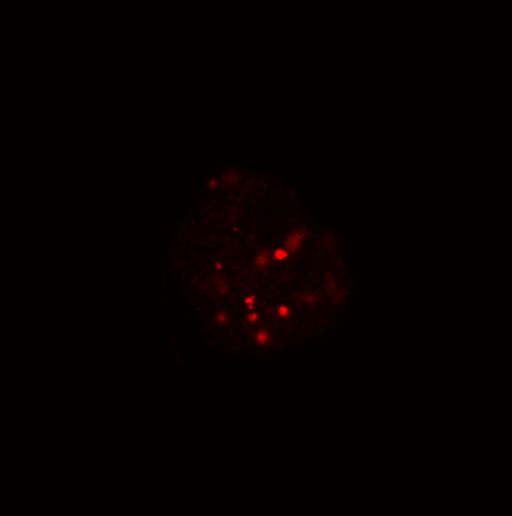

Supplement: Supplementary file 12 — Source data Fig. 2 [file 44318_2026_770_MOESM12_ESM.zip › Figure_2/R287/R287 rfp.tif]

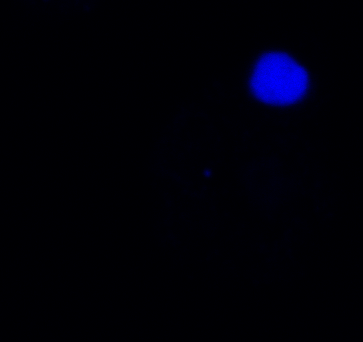

Supplement: Supplementary file 12 — Source data Fig. 2 [file 44318_2026_770_MOESM12_ESM.zip › Figure_2/R317/R317 dapi.tif]

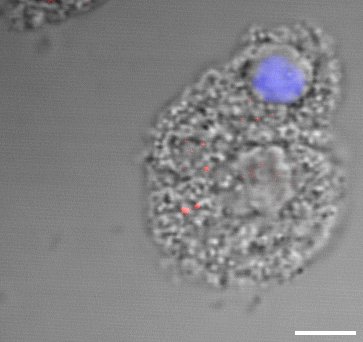

Supplement: Supplementary file 12 — Source data Fig. 2 [file 44318_2026_770_MOESM12_ESM.zip › Figure_2/R317/R317 merge dic.jpg]

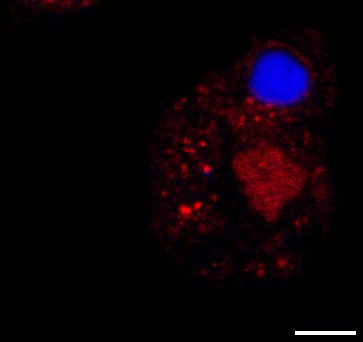

Supplement: Supplementary file 12 — Source data Fig. 2 [file 44318_2026_770_MOESM12_ESM.zip › Figure_2/R317/R317 merge rfp.jpg]

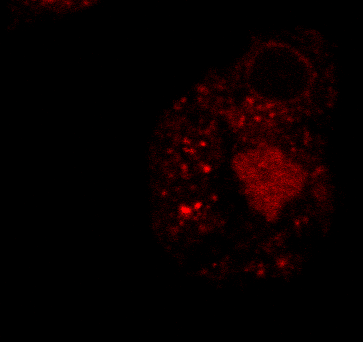

Supplement: Supplementary file 12 — Source data Fig. 2 [file 44318_2026_770_MOESM12_ESM.zip › Figure_2/R317/R317 rfp.tif]

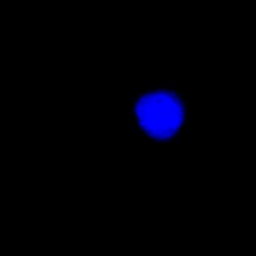

Supplement: Supplementary file 12 — Source data Fig. 2 [file 44318_2026_770_MOESM12_ESM.zip › Figure_2/R347/R347 dapi.tif]

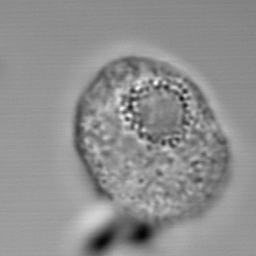

Supplement: Supplementary file 12 — Source data Fig. 2 [file 44318_2026_770_MOESM12_ESM.zip › Figure_2/R347/R347 gray.tif]

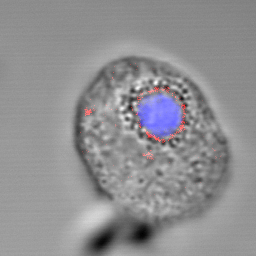

Supplement: Supplementary file 12 — Source data Fig. 2 [file 44318_2026_770_MOESM12_ESM.zip › Figure_2/R347/R347 merge dic.tif]

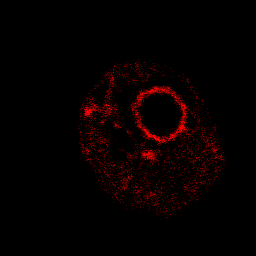

Supplement: Supplementary file 12 — Source data Fig. 2 [file 44318_2026_770_MOESM12_ESM.zip › Figure_2/R347/R347 rfp.tif]

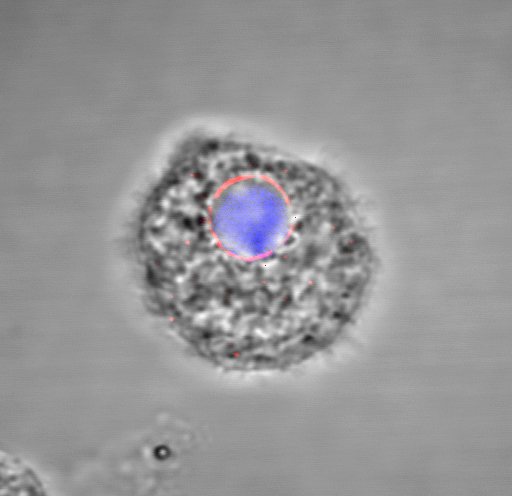

Supplement: Supplementary file 12 — Source data Fig. 2 [file 44318_2026_770_MOESM12_ESM.zip › Figure_2/R387/R387 merge dic.tif]

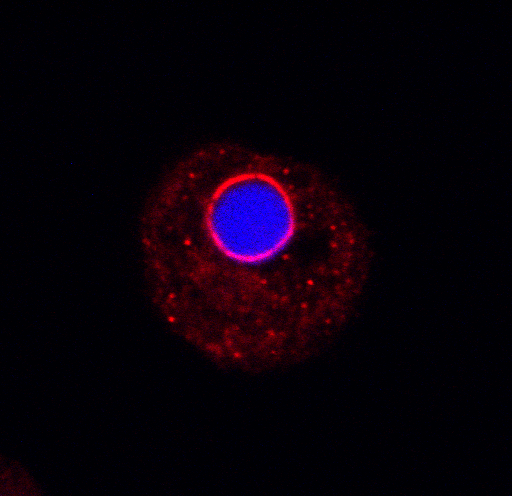

Supplement: Supplementary file 12 — Source data Fig. 2 [file 44318_2026_770_MOESM12_ESM.zip › Figure_2/R387/R387 merge rfp.tif]

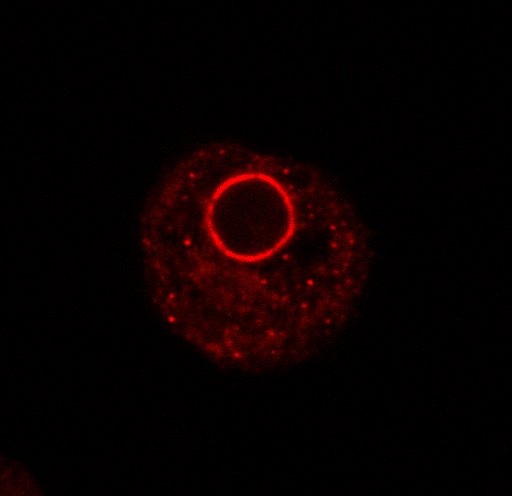

Supplement: Supplementary file 12 — Source data Fig. 2 [file 44318_2026_770_MOESM12_ESM.zip › Figure_2/R387/R387 rfp.tif]

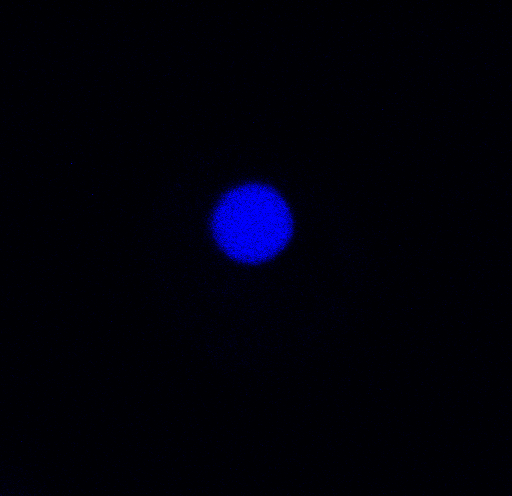

Supplement: Supplementary file 12 — Source data Fig. 2 [file 44318_2026_770_MOESM12_ESM.zip › Figure_2/R387/R387dapi.tif]

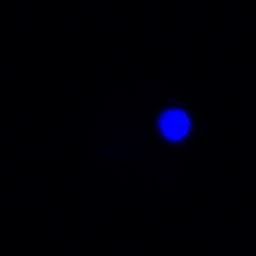

Supplement: Supplementary file 12 — Source data Fig. 2 [file 44318_2026_770_MOESM12_ESM.zip › Figure_2/R443/R443 dapi.tif]

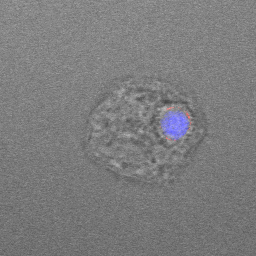

Supplement: Supplementary file 12 — Source data Fig. 2 [file 44318_2026_770_MOESM12_ESM.zip › Figure_2/R443/R443 merge dic.tif]

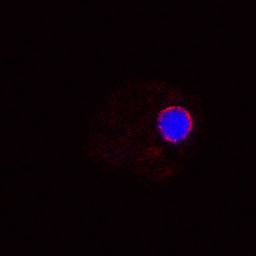

Supplement: Supplementary file 12 — Source data Fig. 2 [file 44318_2026_770_MOESM12_ESM.zip › Figure_2/R443/R443 merge rfp.tif]

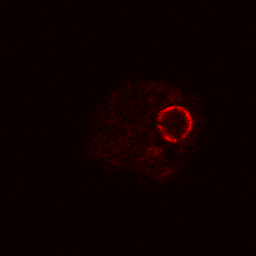

Supplement: Supplementary file 12 — Source data Fig. 2 [file 44318_2026_770_MOESM12_ESM.zip › Figure_2/R443/R443 rfp.tif]

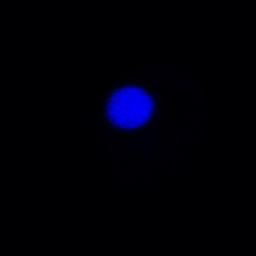

Supplement: Supplementary file 12 — Source data Fig. 2 [file 44318_2026_770_MOESM12_ESM.zip › Figure_2/R513b/R513b dapi.tif]

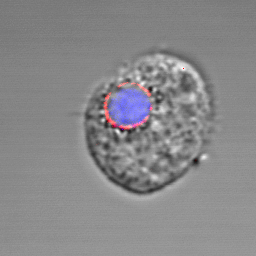

Supplement: Supplementary file 12 — Source data Fig. 2 [file 44318_2026_770_MOESM12_ESM.zip › Figure_2/R513b/R513b merge dic.tif]

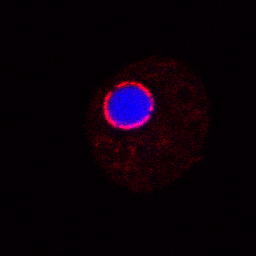

Supplement: Supplementary file 12 — Source data Fig. 2 [file 44318_2026_770_MOESM12_ESM.zip › Figure_2/R513b/R513b merge rfp.tif]

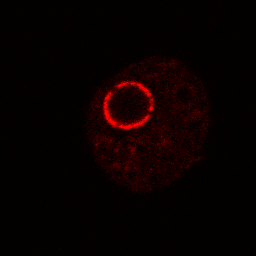

Supplement: Supplementary file 12 — Source data Fig. 2 [file 44318_2026_770_MOESM12_ESM.zip › Figure_2/R513b/R513b rfp.tif]

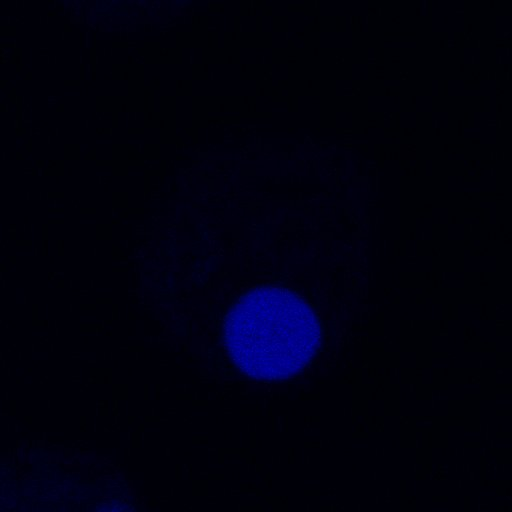

Supplement: Supplementary file 12 — Source data Fig. 2 [file 44318_2026_770_MOESM12_ESM.zip › Figure_2/R595/R595 dapi.tif]

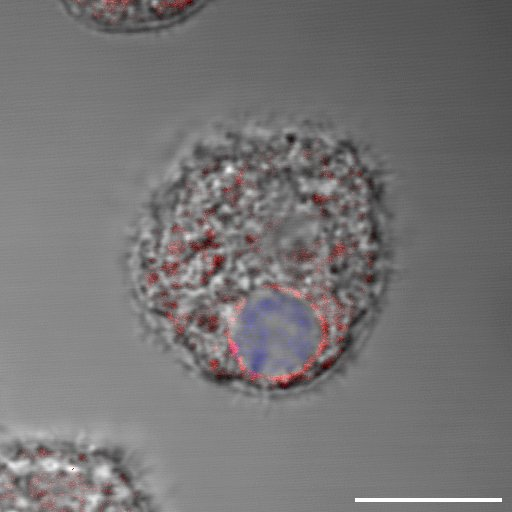

Supplement: Supplementary file 12 — Source data Fig. 2 [file 44318_2026_770_MOESM12_ESM.zip › Figure_2/R595/R595 merge dic.tif]

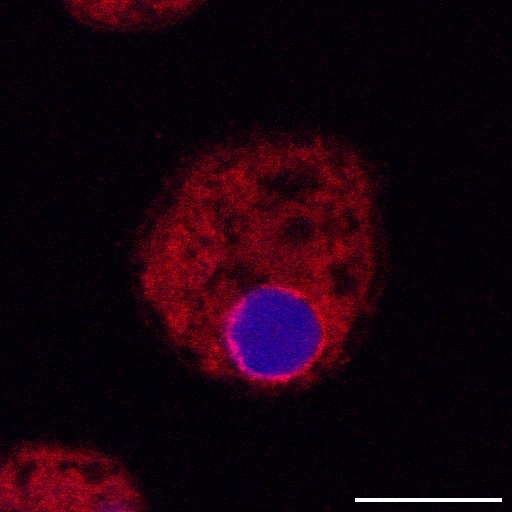

Supplement: Supplementary file 12 — Source data Fig. 2 [file 44318_2026_770_MOESM12_ESM.zip › Figure_2/R595/R595 merge rfp.tif]

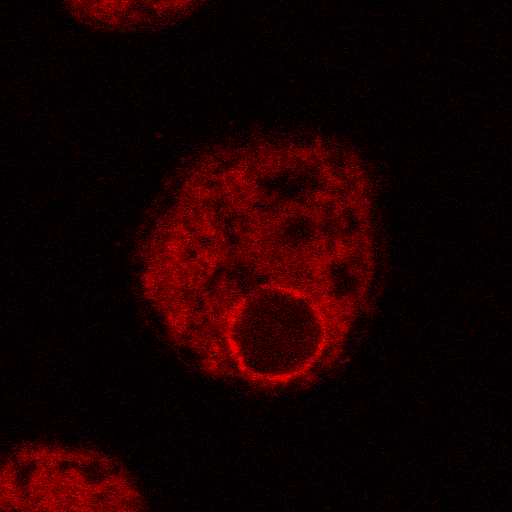

Supplement: Supplementary file 12 — Source data Fig. 2 [file 44318_2026_770_MOESM12_ESM.zip › Figure_2/R595/R595 rfp.tif]

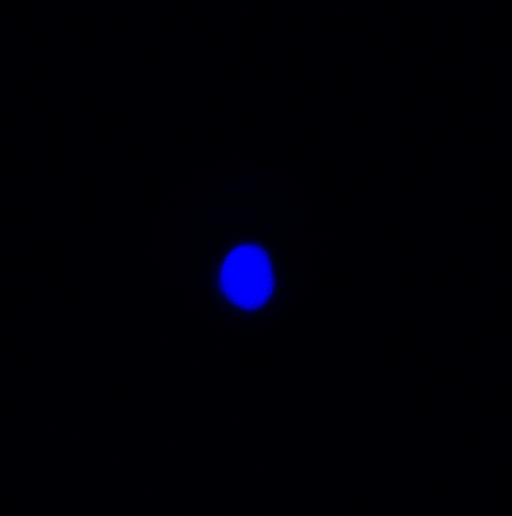

Supplement: Supplementary file 12 — Source data Fig. 2 [file 44318_2026_770_MOESM12_ESM.zip › Figure_2/R710/R710 dapi.tif]

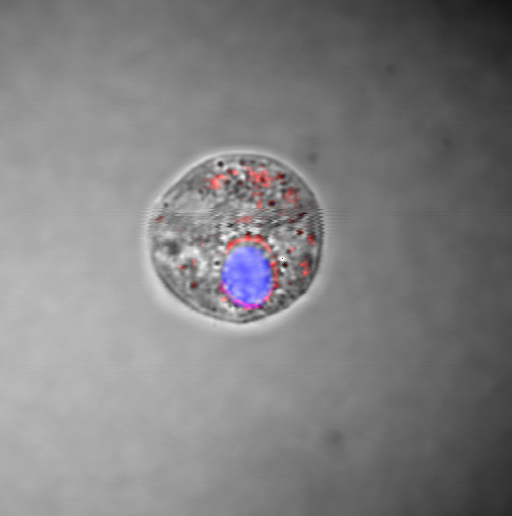

Supplement: Supplementary file 12 — Source data Fig. 2 [file 44318_2026_770_MOESM12_ESM.zip › Figure_2/R710/R710 merge dic.tif]

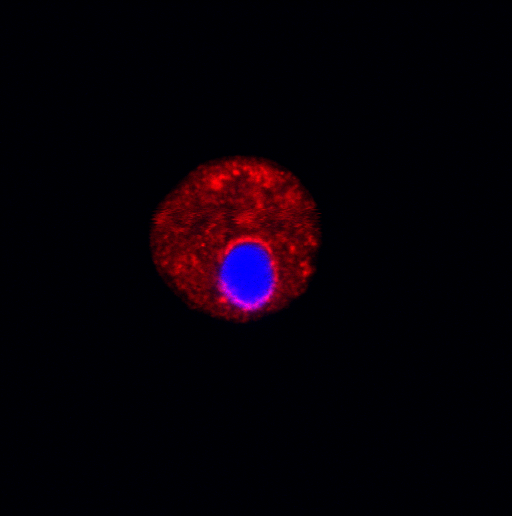

Supplement: Supplementary file 12 — Source data Fig. 2 [file 44318_2026_770_MOESM12_ESM.zip › Figure_2/R710/R710 merge rfp.tif]

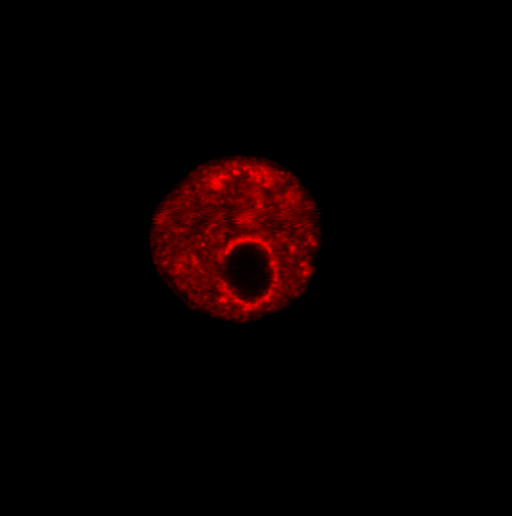

Supplement: Supplementary file 12 — Source data Fig. 2 [file 44318_2026_770_MOESM12_ESM.zip › Figure_2/R710/R710 rfp.tif]

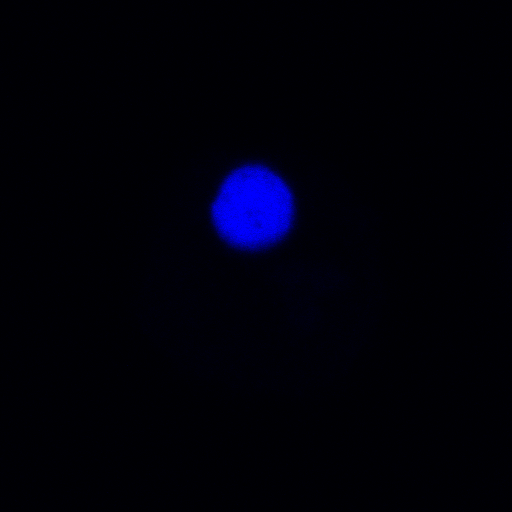

Supplement: Supplementary file 12 — Source data Fig. 2 [file 44318_2026_770_MOESM12_ESM.zip › Figure_2/R721/R721 dapi.tif]

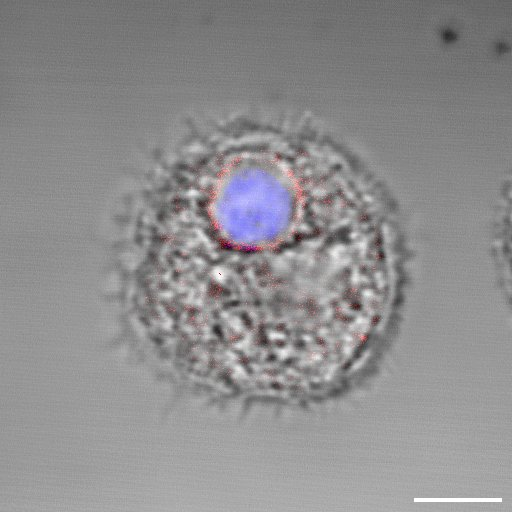

Supplement: Supplementary file 12 — Source data Fig. 2 [file 44318_2026_770_MOESM12_ESM.zip › Figure_2/R721/R721 merge dic.tif]

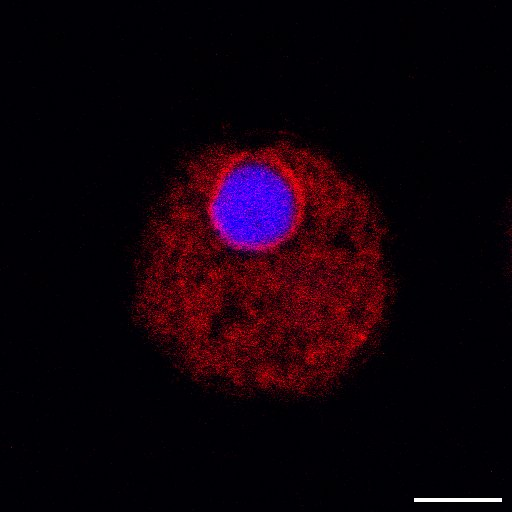

Supplement: Supplementary file 12 — Source data Fig. 2 [file 44318_2026_770_MOESM12_ESM.zip › Figure_2/R721/R721 merge rfp.tif]

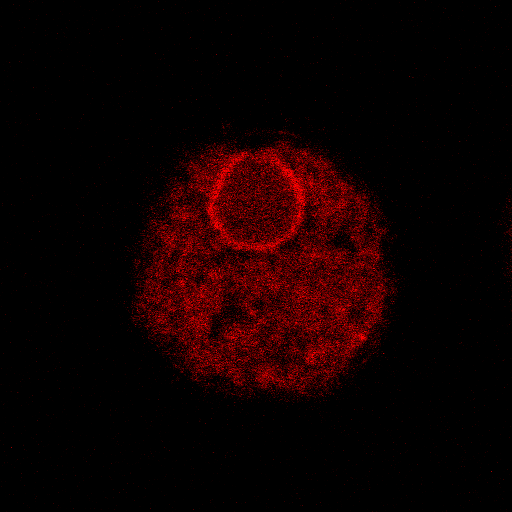

Supplement: Supplementary file 12 — Source data Fig. 2 [file 44318_2026_770_MOESM12_ESM.zip › Figure_2/R721/R721 rfp.tif]

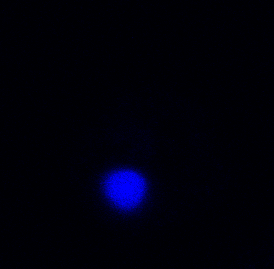

Supplement: Supplementary file 12 — Source data Fig. 2 [file 44318_2026_770_MOESM12_ESM.zip › Figure_2/WT/WT dapi.tif]

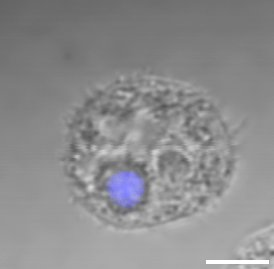

Supplement: Supplementary file 12 — Source data Fig. 2 [file 44318_2026_770_MOESM12_ESM.zip › Figure_2/WT/WT merge dic.tif]

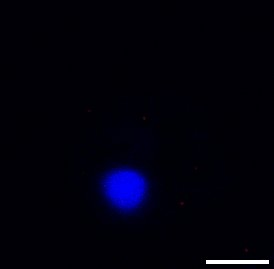

Supplement: Supplementary file 12 — Source data Fig. 2 [file 44318_2026_770_MOESM12_ESM.zip › Figure_2/WT/WT merge rfp.tif]

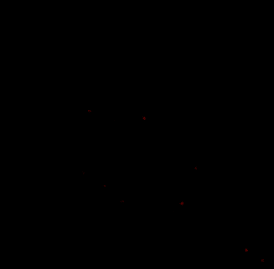

Supplement: Supplementary file 12 — Source data Fig. 2 [file 44318_2026_770_MOESM12_ESM.zip › Figure_2/WT/WT rfp.tif]

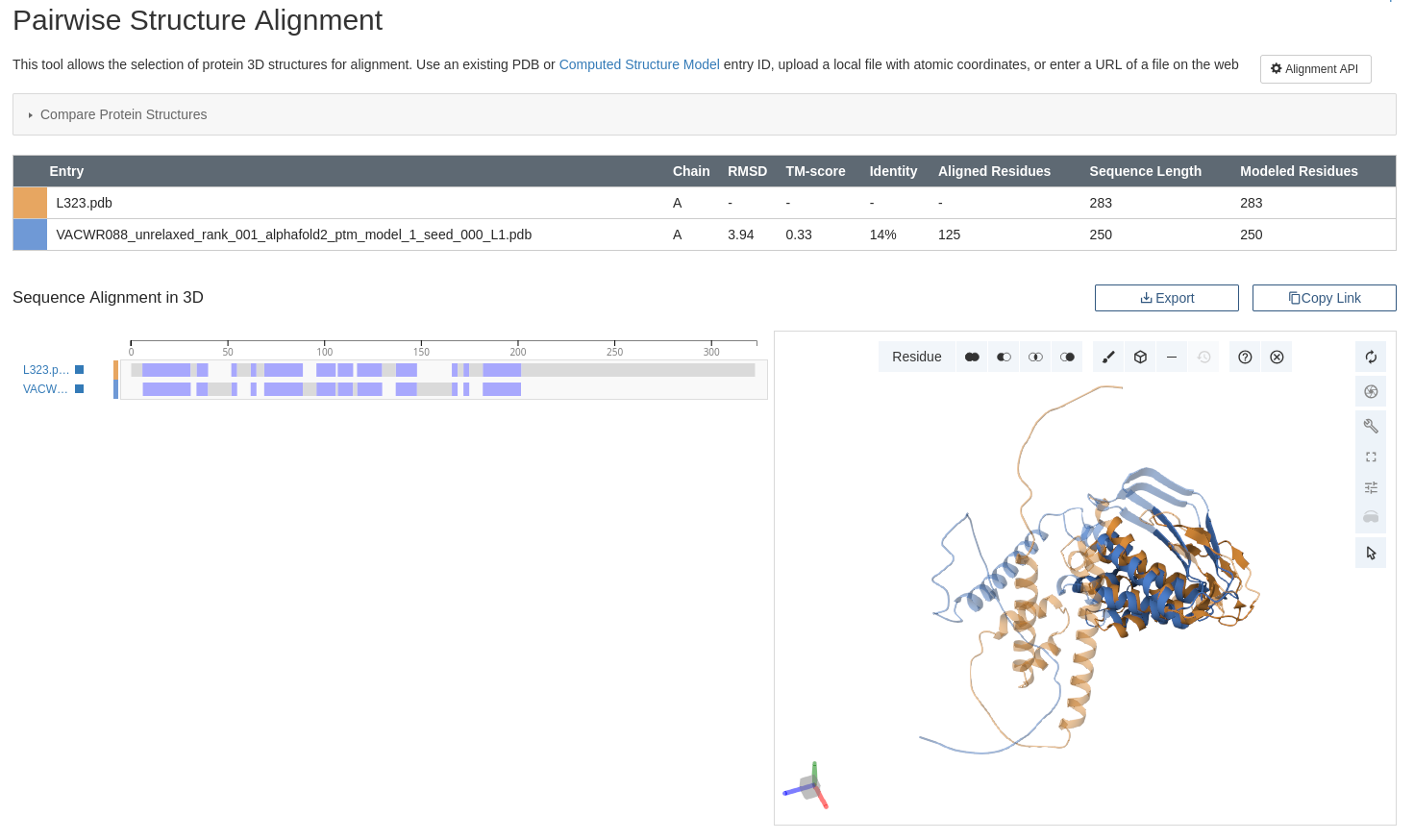

Supplement: Supplementary file 14 — Source data Fig. 4 [file 44318_2026_770_MOESM14_ESM.zip › Figure_4/4B/rigid_body_alignment/rcsb_pairwise_alignment_jce/jce_results_L323_L1_rigid_body.png]

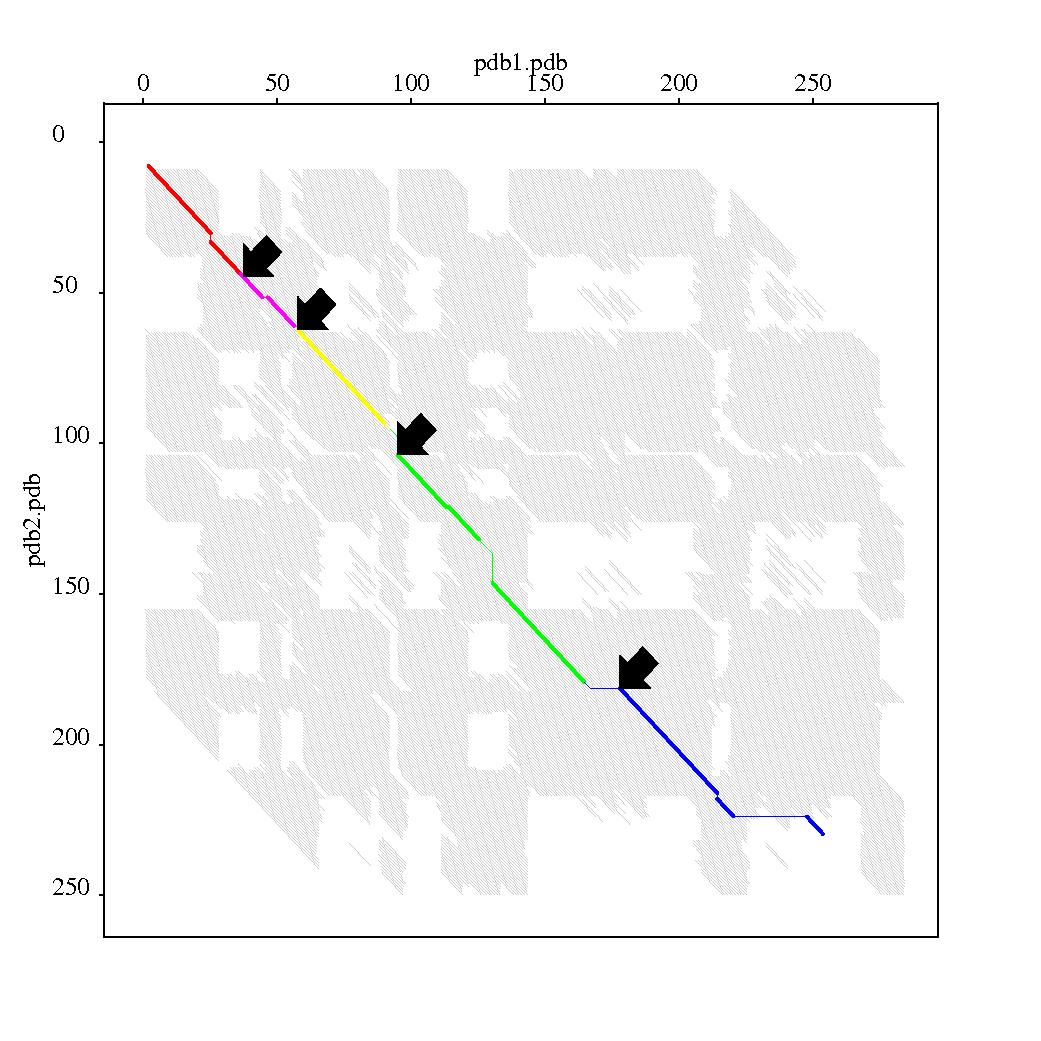

Supplement: Supplementary file 14 — Source data Fig. 4 [file 44318_2026_770_MOESM14_ESM.zip › Figure_4/4B/flexible_alignment/fatcat_aligned_pdbs/pdb1.pdb2.afp.color.jpeg]

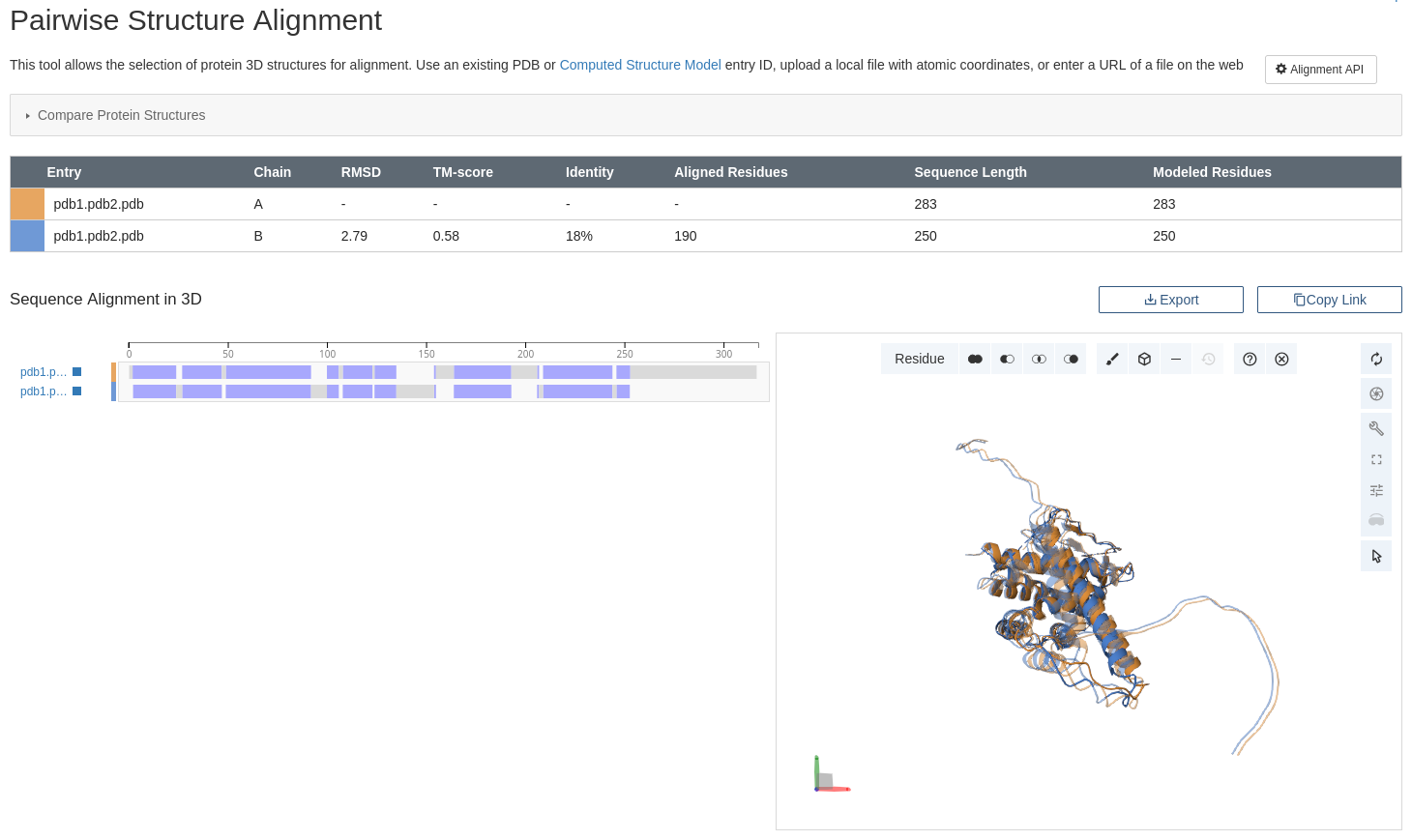

Supplement: Supplementary file 14 — Source data Fig. 4 [file 44318_2026_770_MOESM14_ESM.zip › Figure_4/4B/flexible_alignment/fatcat_rcsb_pairwise_alignment_jce/jce_results_L323_L1_flexible.png]

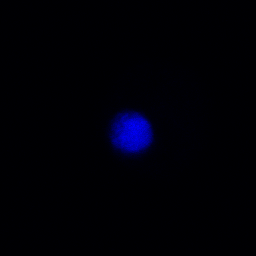

Supplement: Supplementary file 15 — Source data Fig. 5 [file 44318_2026_770_MOESM15_ESM.zip › Figure_5/5C/Microscopy images/Complementation R443_CXXS/Complementation R443_CXXS dapi.tif]

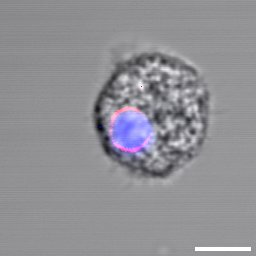

Supplement: Supplementary file 15 — Source data Fig. 5 [file 44318_2026_770_MOESM15_ESM.zip › Figure_5/5C/Microscopy images/Complementation R443_CXXS/Complementation R443_CXXS merge dic.tif]

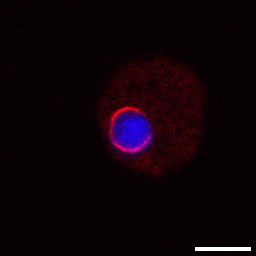

Supplement: Supplementary file 15 — Source data Fig. 5 [file 44318_2026_770_MOESM15_ESM.zip › Figure_5/5C/Microscopy images/Complementation R443_CXXS/Complementation R443_CXXS merge rfp.tif]

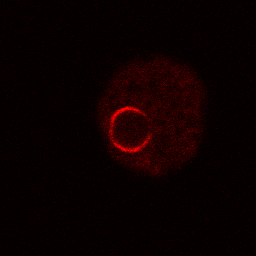

Supplement: Supplementary file 15 — Source data Fig. 5 [file 44318_2026_770_MOESM15_ESM.zip › Figure_5/5C/Microscopy images/Complementation R443_CXXS/Complementation R443_CXXS rfp.tif]

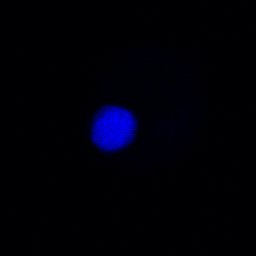

Supplement: Supplementary file 15 — Source data Fig. 5 [file 44318_2026_770_MOESM15_ESM.zip › Figure_5/5C/Microscopy images/Complementation R443_SXXC/Complementation R443_SXXC dapi.tif]

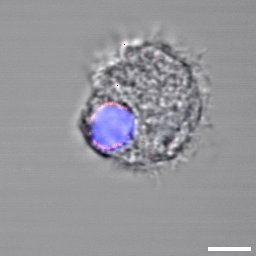

Supplement: Supplementary file 15 — Source data Fig. 5 [file 44318_2026_770_MOESM15_ESM.zip › Figure_5/5C/Microscopy images/Complementation R443_SXXC/Complementation R443_SXXC merge dic.tif]

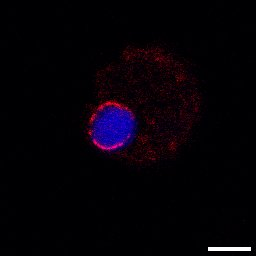

Supplement: Supplementary file 15 — Source data Fig. 5 [file 44318_2026_770_MOESM15_ESM.zip › Figure_5/5C/Microscopy images/Complementation R443_SXXC/Complementation R443_SXXC merge rfp.tif]

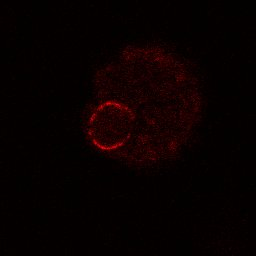

Supplement: Supplementary file 15 — Source data Fig. 5 [file 44318_2026_770_MOESM15_ESM.zip › Figure_5/5C/Microscopy images/Complementation R443_SXXC/Complementation R443_SXXC rfp.tif]

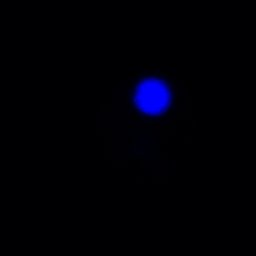

Supplement: Supplementary file 15 — Source data Fig. 5 [file 44318_2026_770_MOESM15_ESM.zip › Figure_5/5C/Microscopy images/Complementation R443_WT/Complementation R443_WT dapi.tif]

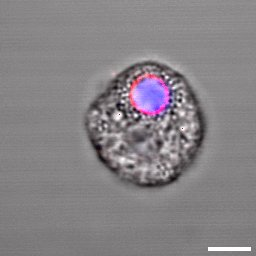

Supplement: Supplementary file 15 — Source data Fig. 5 [file 44318_2026_770_MOESM15_ESM.zip › Figure_5/5C/Microscopy images/Complementation R443_WT/Complementation R443_WT merge dic.tif]

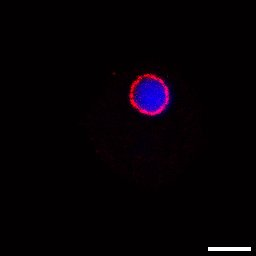

Supplement: Supplementary file 15 — Source data Fig. 5 [file 44318_2026_770_MOESM15_ESM.zip › Figure_5/5C/Microscopy images/Complementation R443_WT/Complementation R443_WT merge rfp.tif]

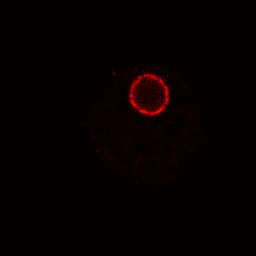

Supplement: Supplementary file 15 — Source data Fig. 5 [file 44318_2026_770_MOESM15_ESM.zip › Figure_5/5C/Microscopy images/Complementation R443_WT/Complementation R443_WT rfp.tif]

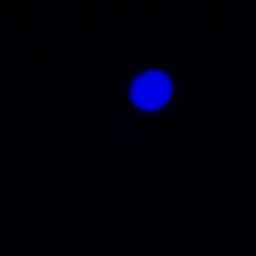

Supplement: Supplementary file 15 — Source data Fig. 5 [file 44318_2026_770_MOESM15_ESM.zip › Figure_5/5C/Microscopy images/R443 KO/R443 KO dapi.tif]
