# Supplementary figures and images for: Elucidating the protein interaction network of one of the largest icosahedral capsids in the virosphere (part 2 of 2)
Source: EMBO J. 2026 Apr 10;45(10):3514–39. doi: 10.1038/s44318-026-00770-8 (PMC13186993; doi:10.1038/s44318-026-00770-8)

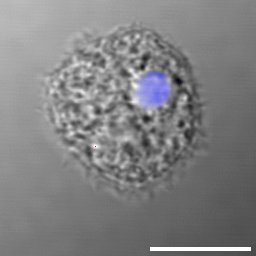

Supplement: Supplementary file 15 — Source data Fig. 5 [file 44318_2026_770_MOESM15_ESM.zip › Figure_5/5C/Microscopy images/R443 KO/R443 KO merge dic.jpg]

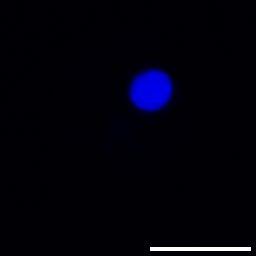

Supplement: Supplementary file 15 — Source data Fig. 5 [file 44318_2026_770_MOESM15_ESM.zip › Figure_5/5C/Microscopy images/R443 KO/R443 KO merge rfp.jpg]

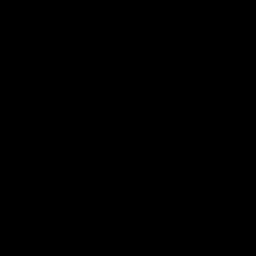

Supplement: Supplementary file 15 — Source data Fig. 5 [file 44318_2026_770_MOESM15_ESM.zip › Figure_5/5C/Microscopy images/R443 KO/R443 KO rfp.tif]

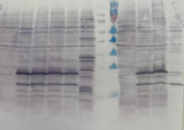

Supplement: Supplementary file 15 — Source data Fig. 5 [file 44318_2026_770_MOESM15_ESM.zip › Figure_5/5C/Western blot/WB anti-HA.tif]

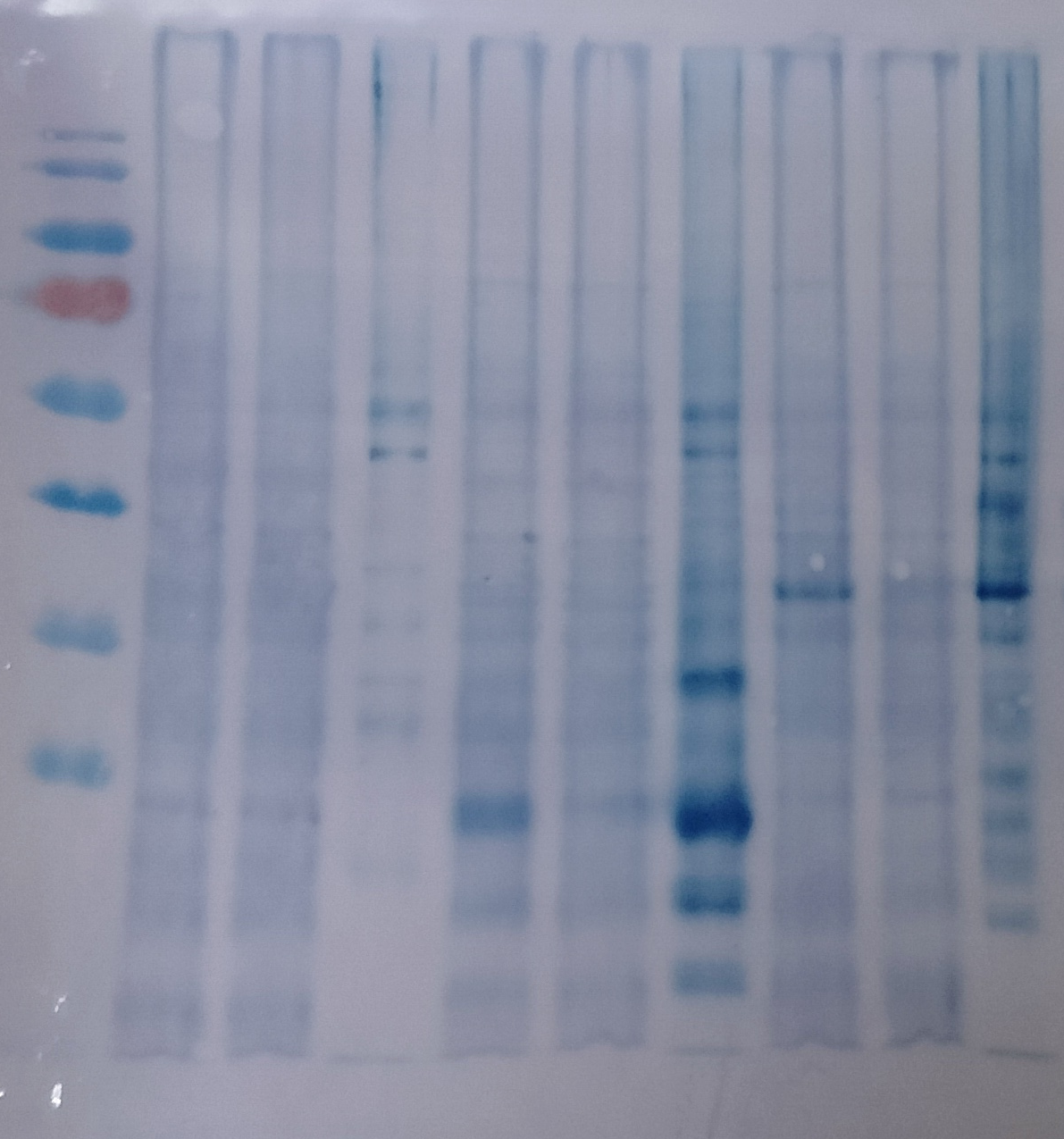

Supplement: Supplementary file 17 — Appendix Figure Source Data [file 44318_2026_770_MOESM17_ESM.zip › Blot 1 WT L443, L330.tif]

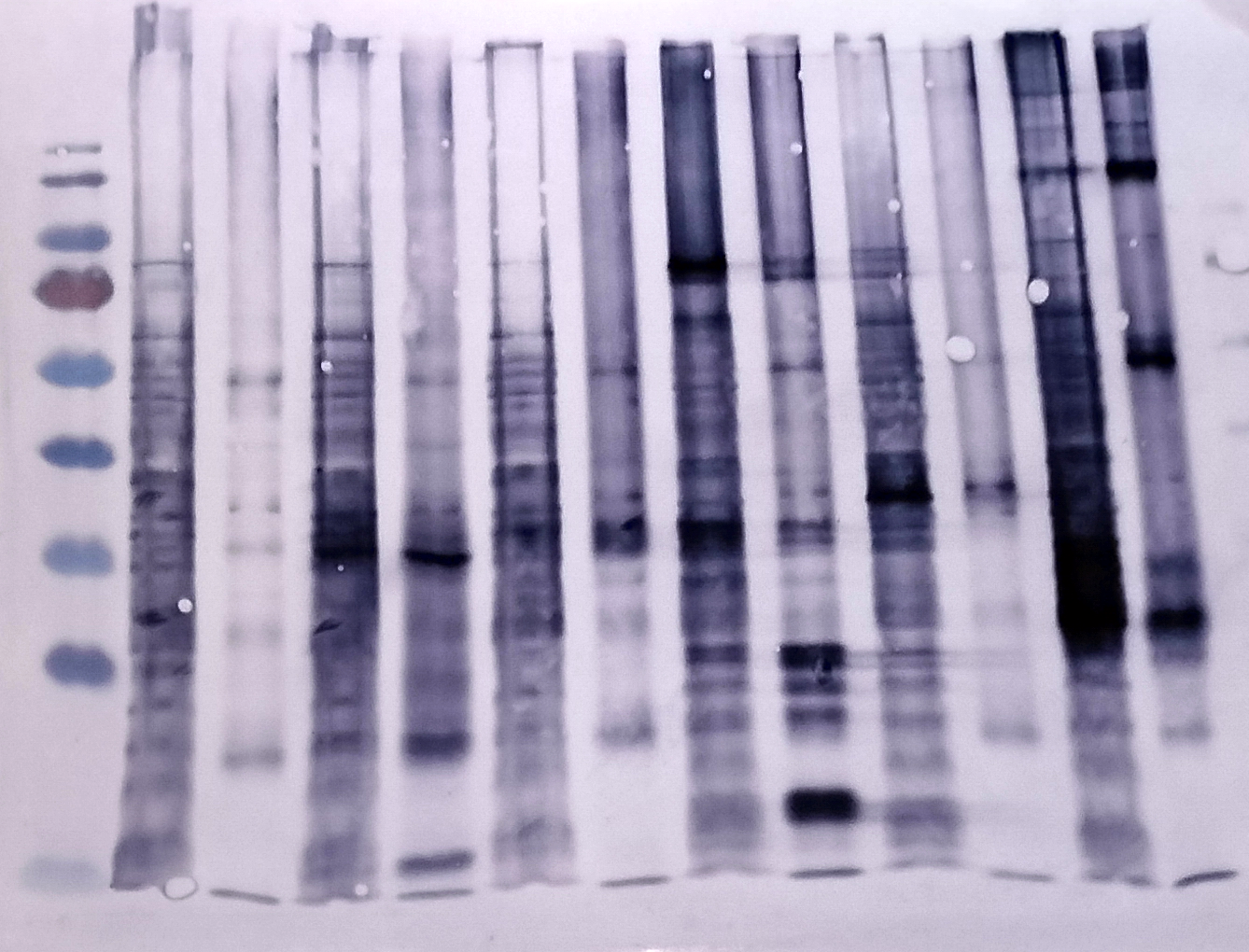

Supplement: Supplementary file 17 — Appendix Figure Source Data [file 44318_2026_770_MOESM17_ESM.zip › Blot 2 WT, L323, R595, R721.tif]

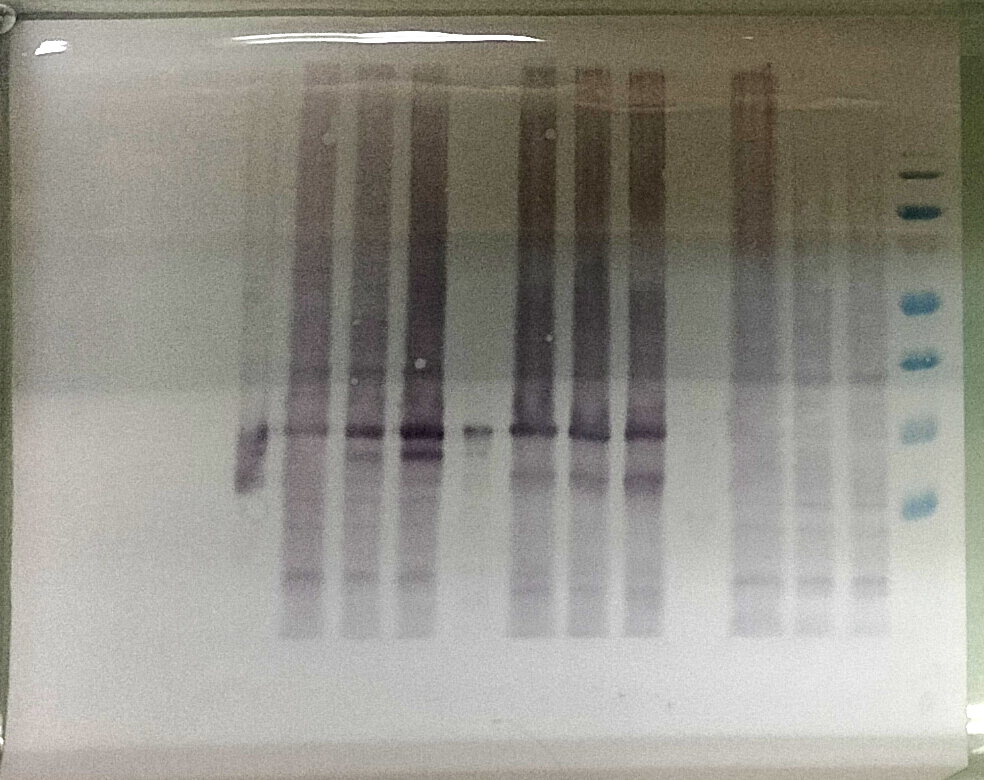

Supplement: Supplementary file 17 — Appendix Figure Source Data [file 44318_2026_770_MOESM17_ESM.zip › Blot 3 WT R317 and L593.tif]

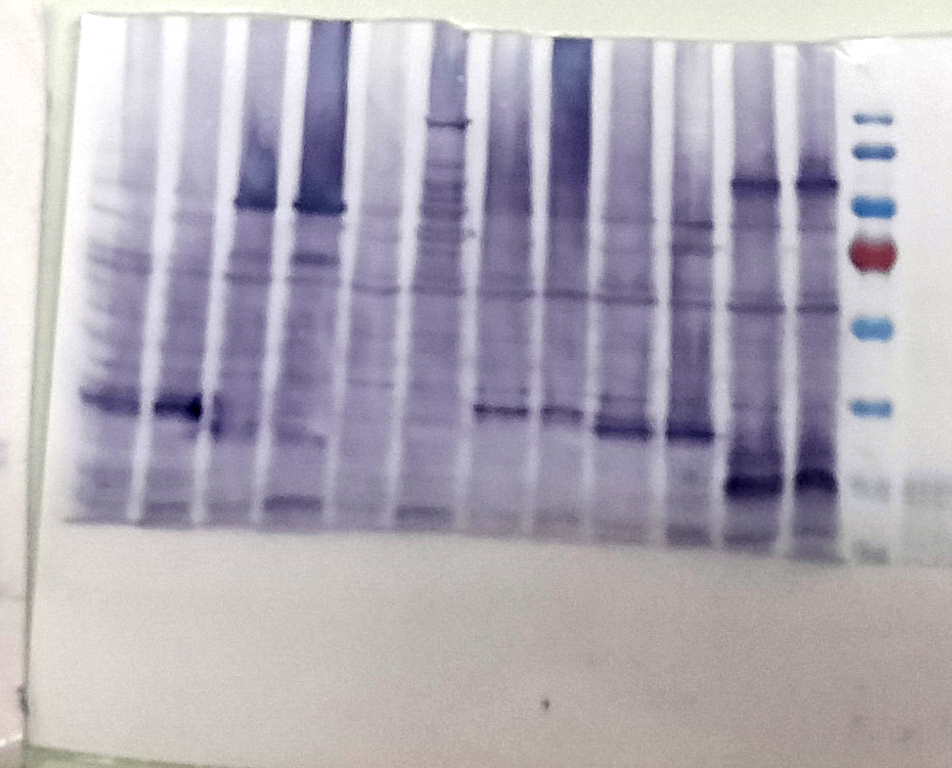

Supplement: Supplementary file 17 — Appendix Figure Source Data [file 44318_2026_770_MOESM17_ESM.zip › Blot 4 WT, L264 and L454 .tif]
