# Supplementary material for: Elucidating the protein interaction network of one of the largest icosahedral capsids in the virosphere
Source: EMBO J. 2026 Apr 10;45(10):3514–39. doi: 10.1038/s44318-026-00770-8 (PMC13186993; doi:10.1038/s44318-026-00770-8)
Supplement: Supplementary file 17 — Appendix Figure Source Data [file 44318_2026_770_MOESM17_ESM.zip › Legend WB data source.pdf]

Blot 1

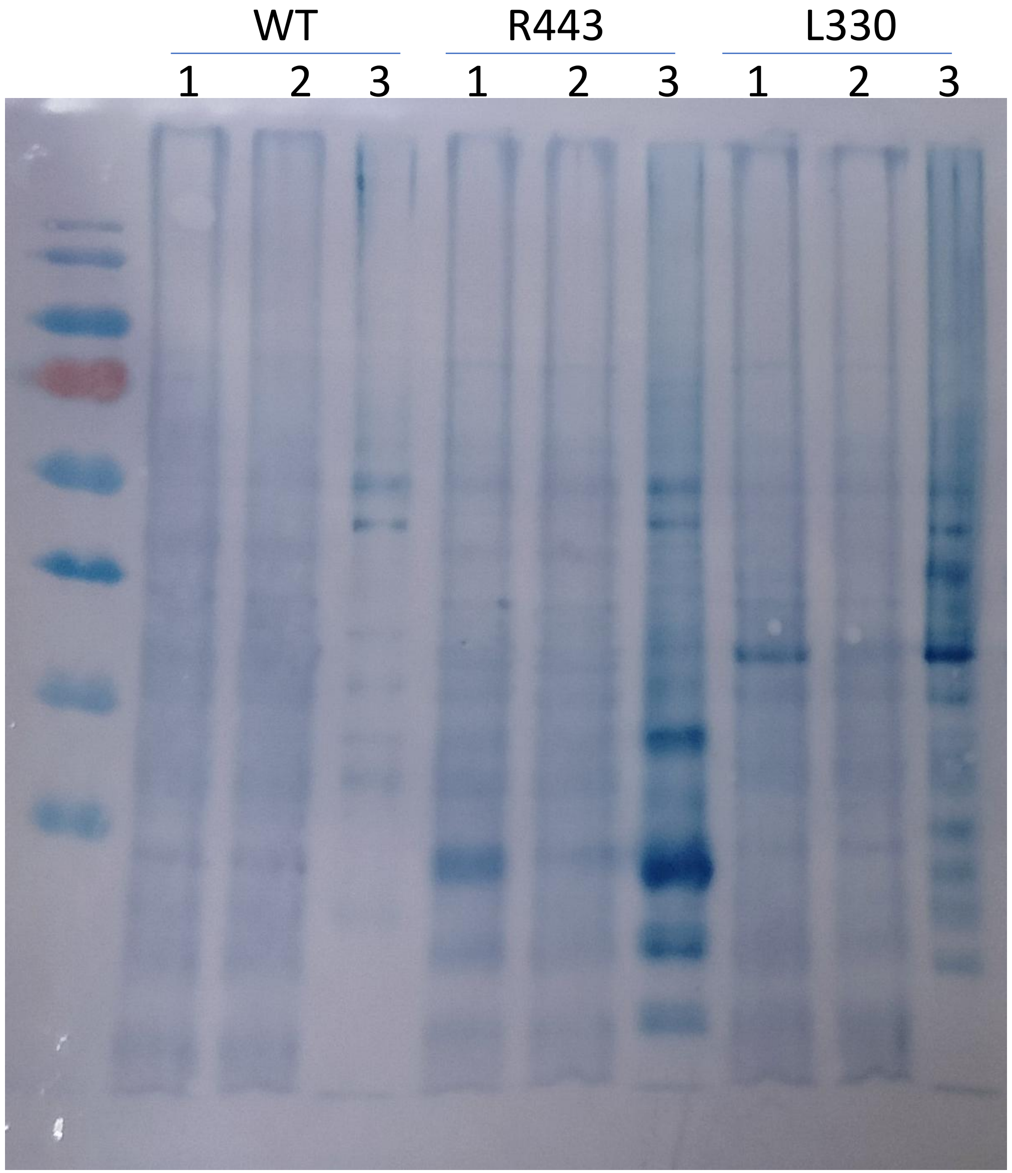

1: Whole extract
2: Supernatant
3: Beads

Blot 2

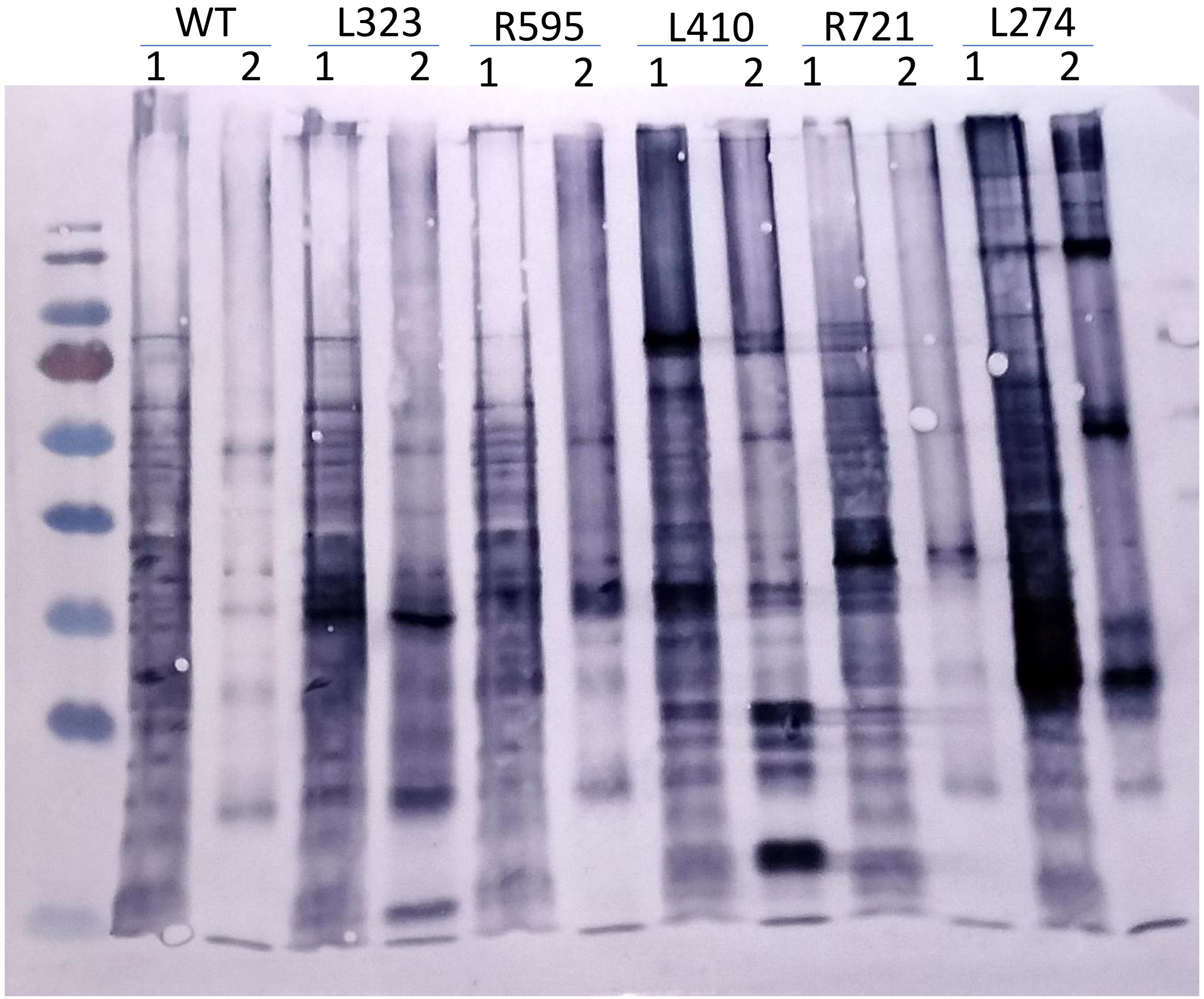

1: Whole extract
2: Supernatant (beads)

Blot 3

WT R317 L593

1 2 3 1 2 3 1 2 3

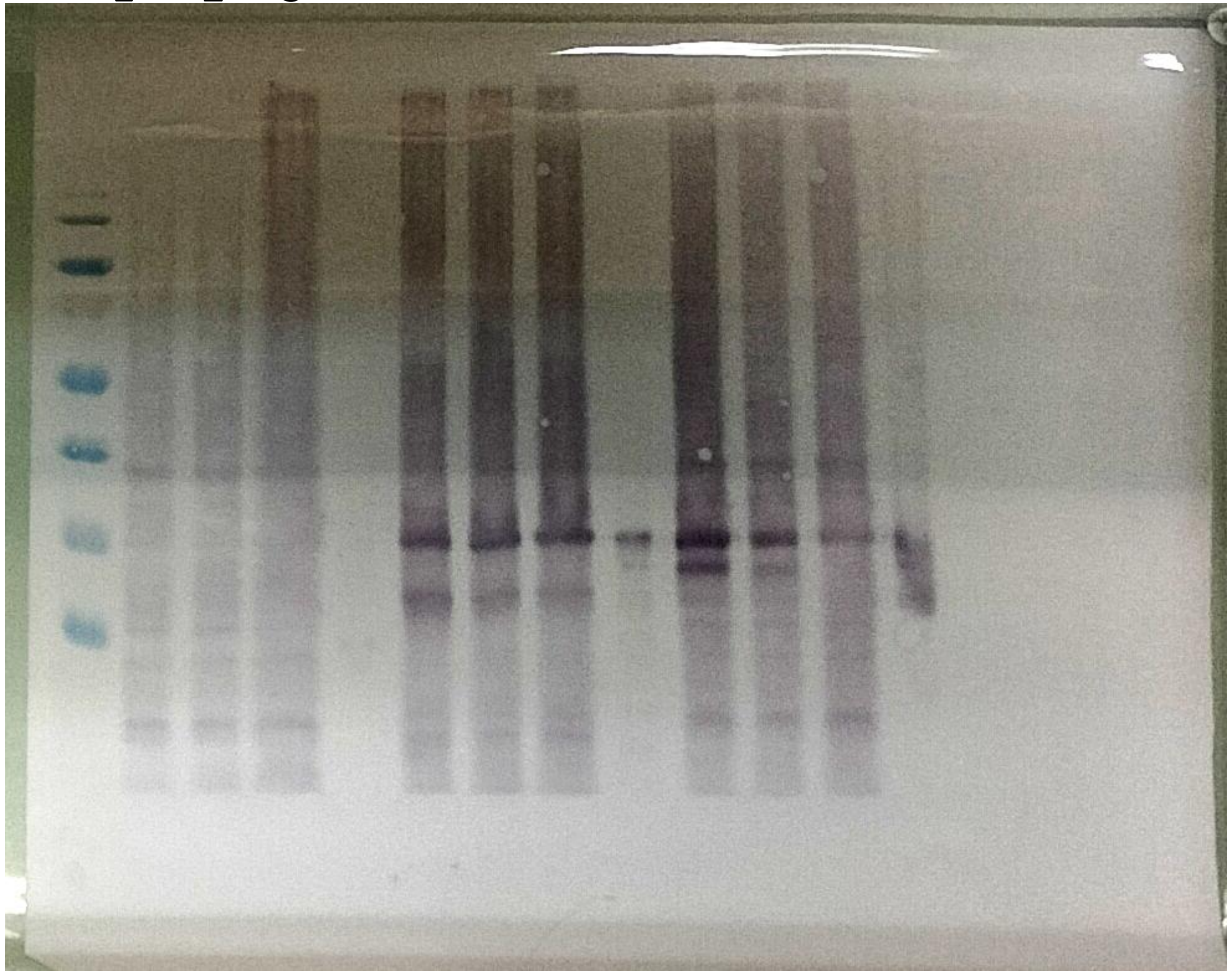

1: Whole extract
2: Supernatant
3: Beads

Blot 4

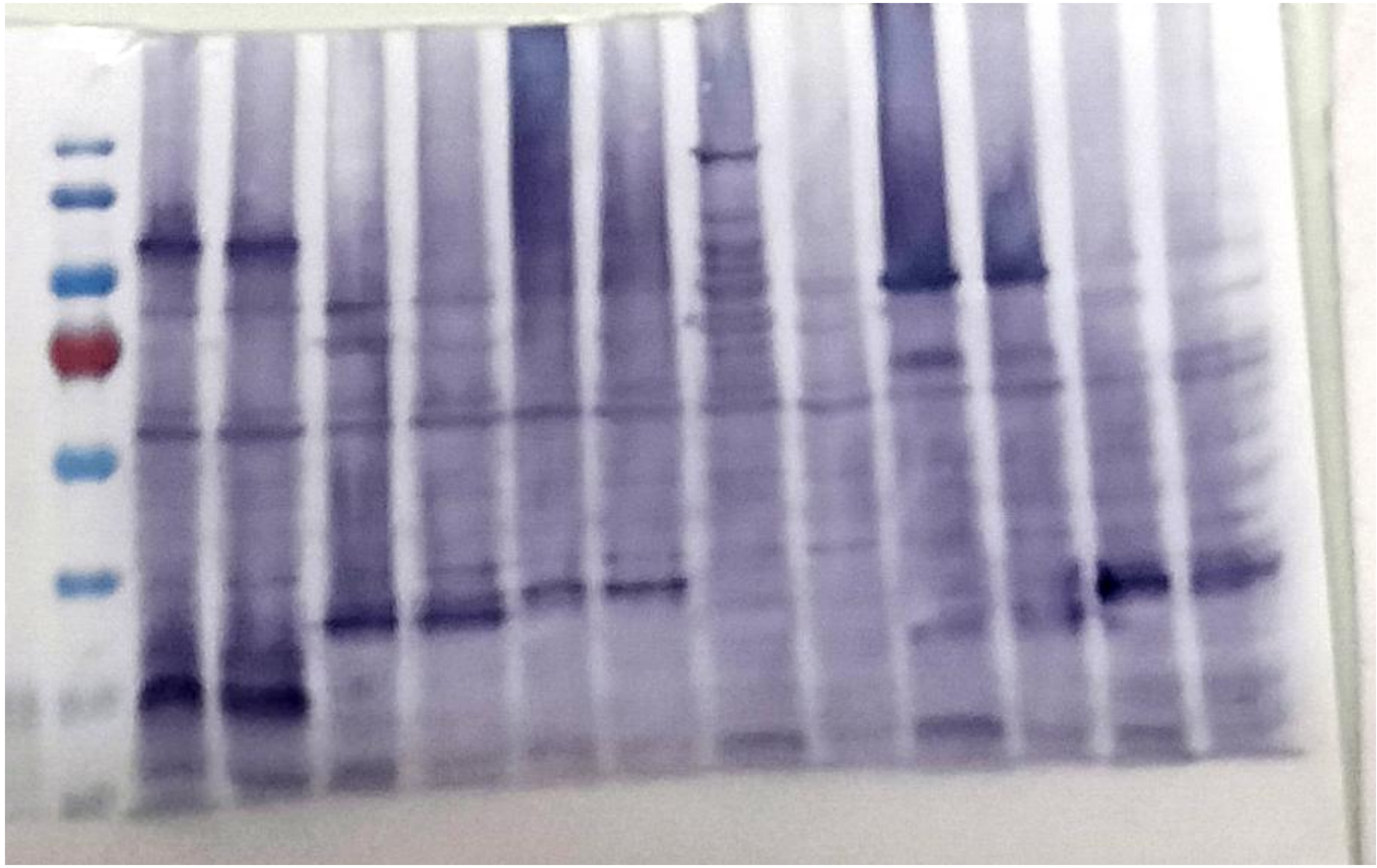

1: Supernatant
