# Supplementary material for: Elucidating the protein interaction network of one of the largest icosahedral capsids in the virosphere
Source: EMBO J. 2026 Apr 10;45(10):3514–39. doi: 10.1038/s44318-026-00770-8 (PMC13186993; doi:10.1038/s44318-026-00770-8)
Supplement: Supplementary file 18 — Expanded View Figures [file 44318_2026_770_MOESM18_ESM.pdf]

# Expanded View Figures

**Figure EV1. Mimivirus AF models in clusters containing poxvirus and ASFV RNA polymerase PDB complexes.**

(**A**) Poxvirus 6RFL RNA polymerase PDB complex and mimivirus homologous AlphaFold3 model. Only the 7 subunits with mimivirus homologs are shown. (**B**) Poxvirus 6RFL_J structure and superimposed mimivirus R357b AF model. Pymol cealign method led to an RMSD of 2.21 Å over 48 residues. (**C**) Summary table of the 6RFL and mimivirus homologous proteins with sequence or structure similarity *e* value. (**D**) Clustering merge step strategy illustrated by 6RFL_J and R357b clusters connections, based on structural similarity matches (Foldseek c >70% and TM-score>0.4) with > 65% of the other cluster. (**E**) Comparison of the LCA taxonomic levels for all clusters containing a mimivirus member, with or without the merge step. The merge step correction reduces the overall cluster count from 690 to 613, lowering the subfamily level cluster count while increasing the superkingdom taxonomic level counts.

 © The Author(s)

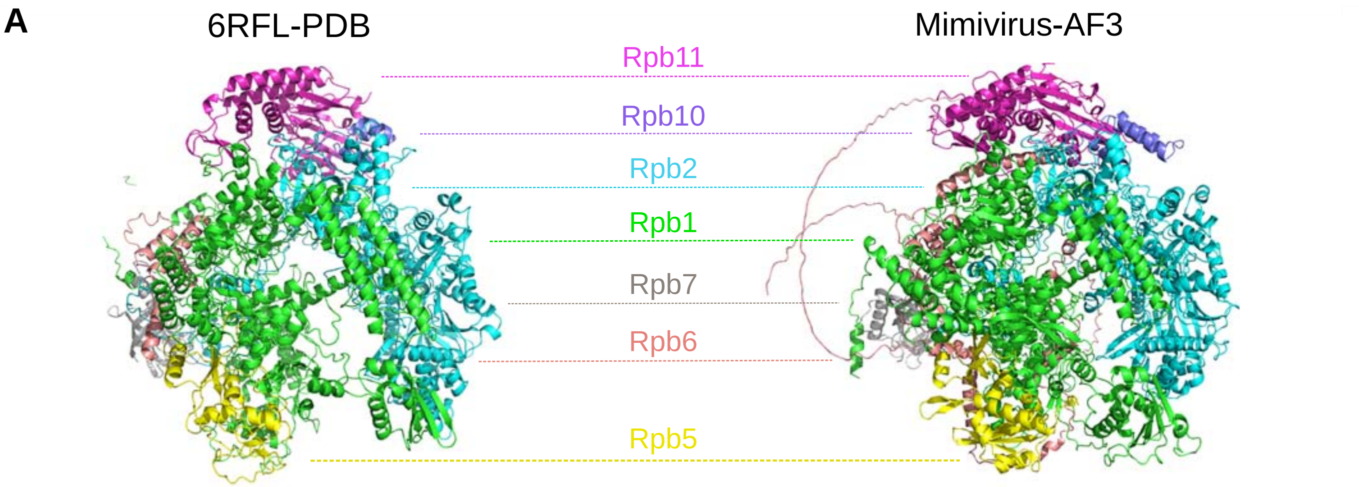


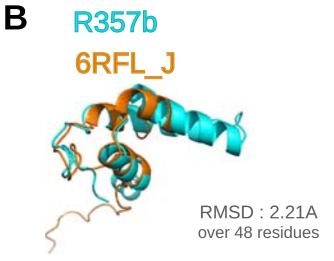


**C** 6RFL - Mimivirus RNA polymerase subunits

| DNA Directed Polymerase subunit | 6RFL-PDB | Mimivirus | Blast E-value | Foldseek E-value |
|---|---|---|---|---|
| **Rpb1** | 6RFL_A | **R501** | 2.00E-46 | |
| **Rpb2** | 6RFL_B | **L244** | 3.00E-68 | |
| **Rpb11** | 6RFL_C | **R470** | - | 1.98E-07 |
| **Rpb5** | 6RFL_E | **L235** | - | 4.83E-06 |
| **Rpb6** | 6RFL_F | **R209** | - | 4.24E-02 |
| **Rpb7 Nter** | 6RFL_G | **L376** | - | 1.37E-09 |
| **Rpb10** | 6RFL_J | **R357b** | - | 1.23E-01 |

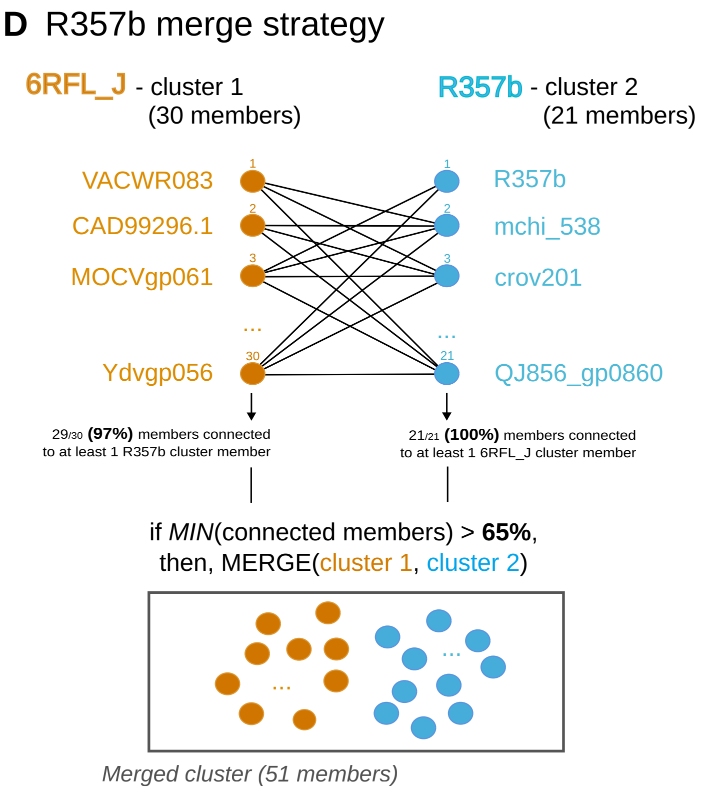


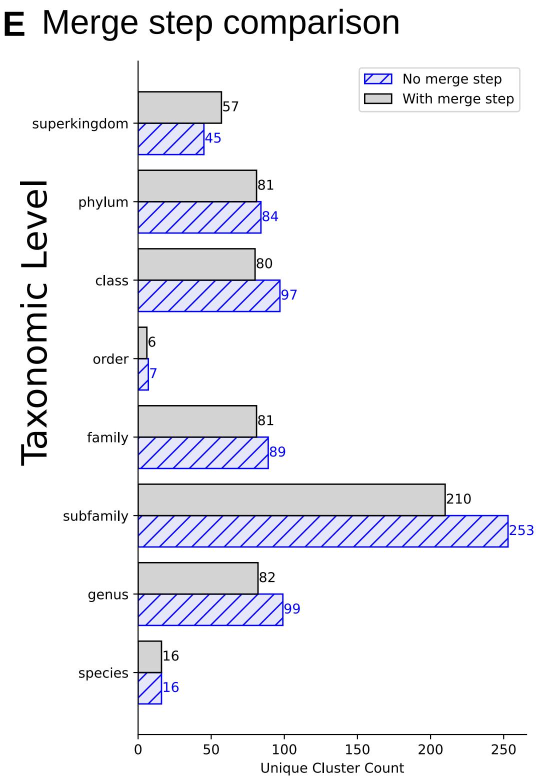

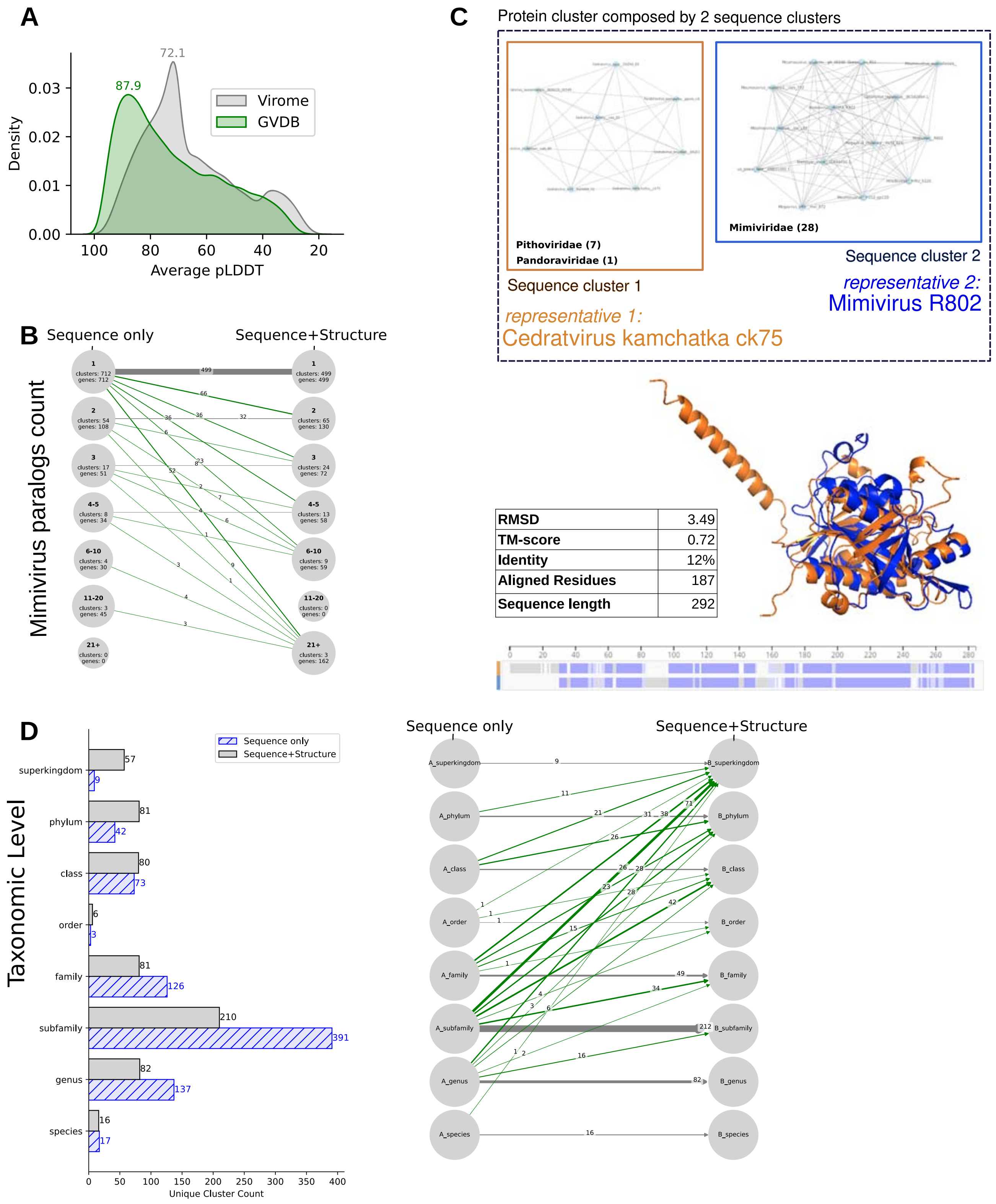


**Figure EV2. Clustering enhancement combining sequence and 3D AF models.**

(**A**) Distribution of the pLDDT scores of AF models in the Virome and GVDB databases. (**B**) Distribution of the Number of paralogs in mimivirus, identified by Sequence-only or Sequence+Structure clustering strategy. (**C**) Example of 2 sequence clusters (*Pithoviridae/Pandoraviridae* and *Mimiviridae*) merged using sequence+structure clustering (TM-score 0.72 and 12% sequence identity). (**D**) LCA taxonomic levels for mimivirus protein clusters with Sequence-only or Sequence+structure clustering.

 © The Author(s)

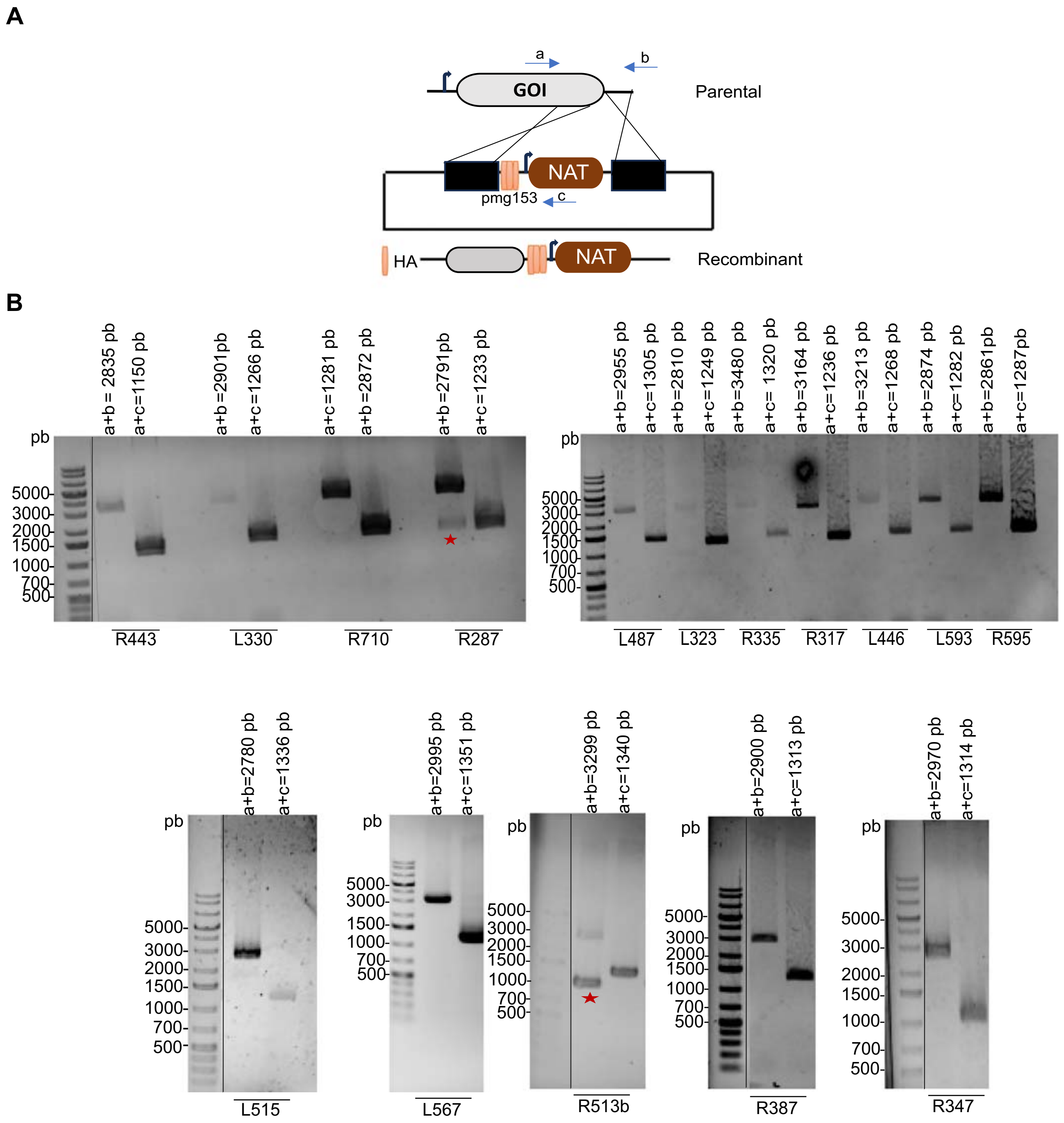


**Figure EV3. Generation of mimivirus recombinant viruses by homologous recombination.**

(**A**) Schematic representation of the vector and strategy used for endogenous HA tagging of mimivirus proteins. GOI: gene of interest. NAT: nourseothricin N-acetyl transferase selection cassette. Primer annealing locations are shown. (**B**) Confirmation of the clonality of mutants by PCR. Expected sizes are indicated in the figure: primers (a + b) used for genotyping on parental viruses and (a + c) for genotyping on recombinant viruses. Red stars highlight non-pure mutant clones.

© The Author(s) 

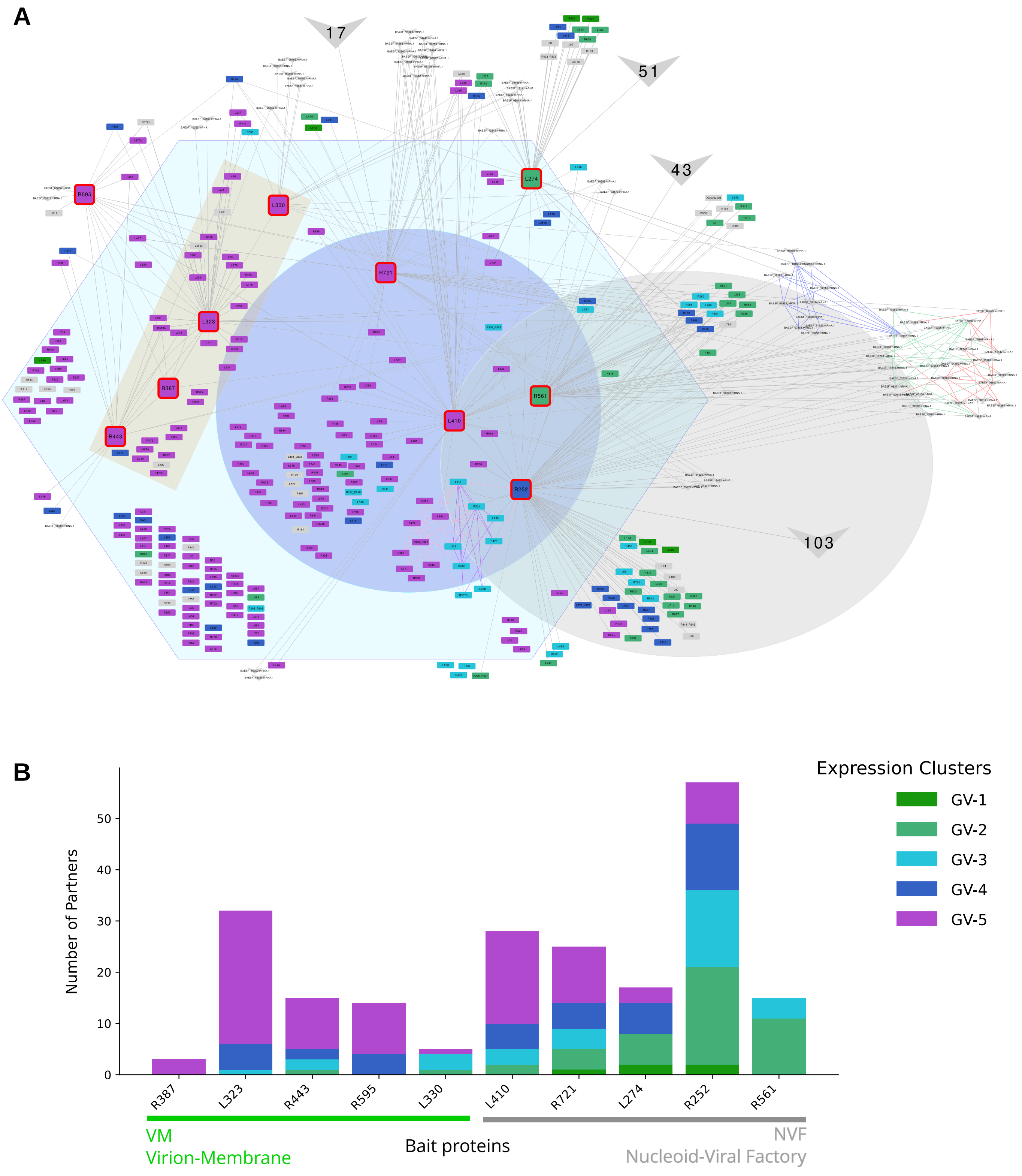


**Figure EV4. Functional partitioning of the VM and NVF subnetworks mirroring temporal expression patterns.**

(**A**) Detailed network showing all connections of the IP-MS baits with their connected proteins. Expression cluster is shown as node colors from green (early genes, GV-1) to violet (late genes, GV-5). (**B**) Expression cluster counts for each IP-MS bait.


© The Author(s)

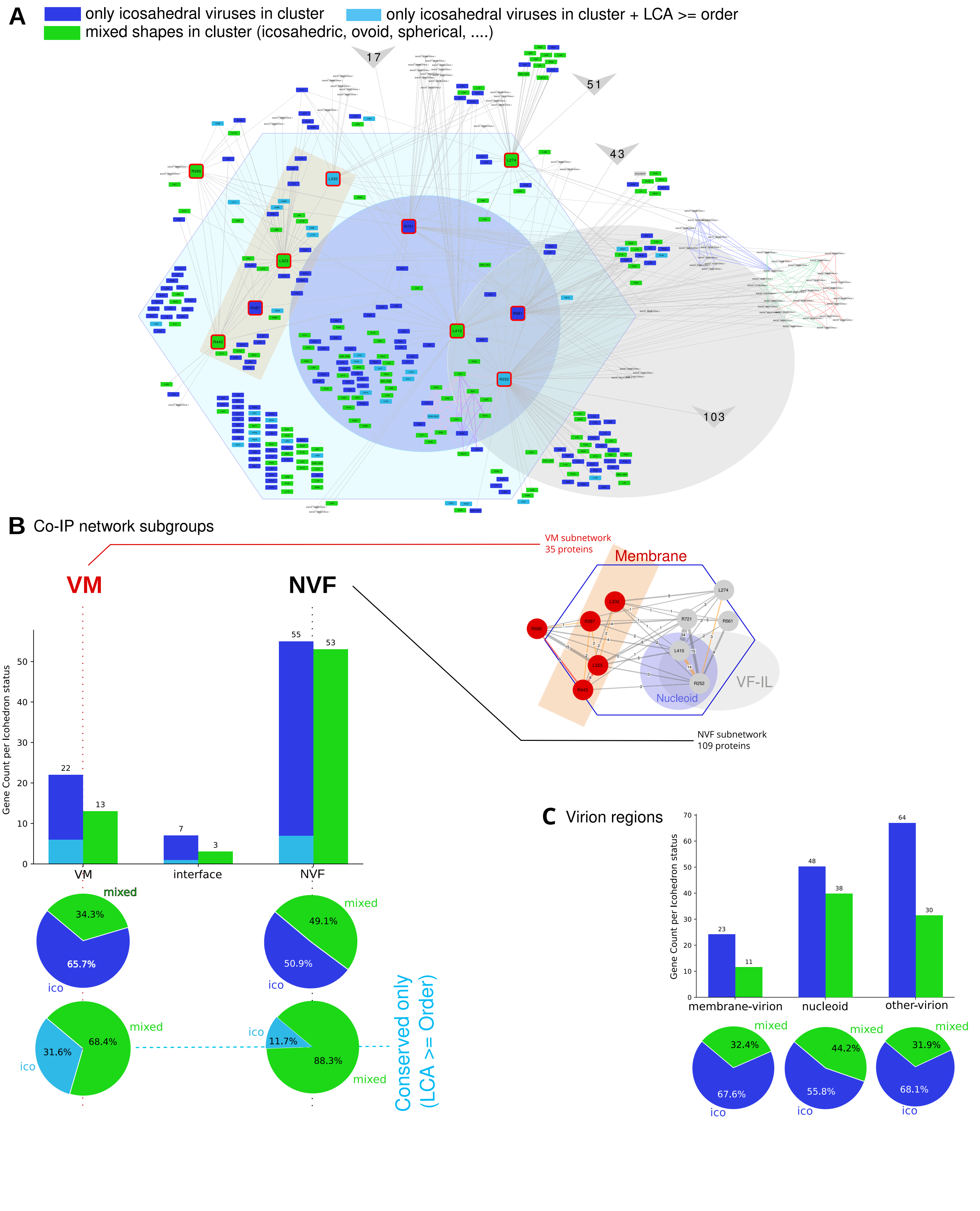
A
only icosahedral viruses in cluster
only icosahedral viruses in cluster + LCA >= order
mixed shapes in cluster (icosahedric, ovoid, spherical, ....)
17
51
43
103
B Co-IP network subgroups
VM subnetwork
35 proteins
Membrane
VM
NVF
VF-IL
Nucleoid
NVF subnetwork
109 proteins
Gene Count per Icohedron status
VM
interface
NVF
mixed
ico
34.3%
65.7%
49.1%
50.9%
68.4%
31.6%
11.7%
88.3%
Conserved only
(LCA >= Order)
C Virion regions
membrane-virion
nucleoid
other-virion
32.4%
67.6%
44.2%
55.8%
31.9%
68.1%

© The Author(s)

◀ **Figure EV5. Co-IP network analysis and virion shape.**

(**A**) Distribution of clusters with varying virion shapes as shown by node colors. Mimivirus proteins belonging to a cluster composed of icosahedral viruses are shown in blue. Light blue proteins indicate that the protein is at least conserved at the order level. Green nodes indicate proteins that belong to clusters enclosing mixed shapes viruses. (**B**) For each sub-network, clusters enclosing proteins from icosahedral-shaped viruses are in blue, and clusters enclosing mixed-shaped viruses are in green. The membrane (VM) sub-network shows a higher proportion of clusters from icosahedral-shaped viruses. (**C**) For each region, independently from the co-IP network, clusters with proteins from icosahedral-shaped viruses are in blue, and clusters enclosing mixed-shaped viruses are in green. The nucleoid region contains a higher fraction of mixed shape protein clusters than the membrane-virion region or the other-virion regions.

 © The Author(s)
